# Effectiveness of the Components of a Digital Multiple Health Behavior Intervention Among University Students (Buddy): Factorial Randomized Trial

**DOI:** 10.2196/88884

**Published:** 2026-03-09

**Authors:** Katarina Åsberg, Oskar Lundgren, Hanna Henriksson, Pontus Henriksson, Ann Catrine Eldh, Preben Bendtsen, Marie Löf, Marcus Bendtsen

**Affiliations:** 1 Department of Health, Medicine and Caring Sciences Linköping University Linköping, Östergötland Sweden; 2 Crown Princess Victoria Children’s Hospital Linköping University Hospital Linköping Sweden; 3 Unit for Strategic Healthcare Region Östergötland Linköping Sweden; 4 Department of Public Health and Caring Sciences Uppsala University Uppsala Sweden; 5 Medical Specialist Clinic Motala Hospital Motala Sweden; 6 Department of Medicine Huddinge Karolinska Institutet Stockholm Sweden

**Keywords:** behavior change, digital interventions, multiple lifestyle, public health, mHealth intervention

## Abstract

**Background:**

Digital interventions have shown promise in supporting healthy behaviors among university students; however, few interventions support simultaneous change across multiple health behaviors. Moreover, behavioral interventions are typically evaluated as a whole, making it challenging to disentangle the contribution of individual components to the overall effects.

**Objective:**

This study estimated the effects of the components of a digital behavior intervention on alcohol, diet, physical activity, and smoking outcomes among university students in Sweden.

**Methods:**

A double-blind randomized factorial trial with 6 two-level factors was conducted. University students in Sweden were proactively recruited through student health care centers and social media. Participants were eligible if they were aged 18 years or older and had at least one health behavior classified as unhealthy. The effects of 6 components were estimated: screening and feedback; goal-setting and planning; motivation; skills and know-how; mindfulness; and self-authored SMS text messages. Primary outcomes were weekly alcohol consumption and frequency of heavy episodic drinking, average daily fruit and vegetable consumption, weekly sugary drink consumption, weekly moderate-to-vigorous physical activity (MVPA), and 4-week point prevalence of smoking.

**Results:**

A total of 1704 students were randomized. The effectiveness of individual and pairwise components was estimated using available data from 1118 (65.61%) participants at 2 months and 874 (51.29%) at 4 months, with sensitivity analyses conducted using imputed missing data. Most consistently, the evidence indicated that screening and feedback affected fruit and vegetable consumption (2-month mean difference 0.11, compatibility interval [CoI] –0.02 to 0.24; probability of effect [POE] 94.7% and 4-month mean difference 0.12, CoI –0.03 to 0.26; POE 94.4%), as did skills and know-how (2-month mean difference 0.19, CoI 0.06-0.33; POE 99.8% and 4-month mean difference 0.14, CoI 0.01-0.28; POE 96.9%). The combination of these 2 components was even more effective (2-month mean difference 0.30, CoI 0.11-0.48; POE 99.9% and 4-month mean difference 0.26, CoI 0.05-0.46; POE 99.4%). The motivation and mindfulness components, both individually and in combination, increased MVPA at 2 months (combined mean difference 78.0, CoI 28.3-128.2; POE 99.9%); however, this effect was not observed at 4 months. Combining screening and feedback with skills and know-how increased MVPA at 4 months (mean difference 60.1, CoI 3.6-116.5; POE 98.2%). Heavy episodic drinking was reduced at 2 months by screening and feedback (incidence rate ratio 0.87, CoI 0.74-1.02; POE 95.2%), and the effect was greater when combined with goal-setting and mindfulness. There was some evidence that the motivation component was harmful with respect to heavy episodic drinking and that self-authored SMS text messages were harmful with respect to sugary drink consumption.

**Conclusions:**

We dismantled a complex digital multiple behavior intervention and examined it using a factorial design to provide novel insights into the effectiveness of the intervention’s different components. Both marginal and synergistic effects were observed across multiple behaviors, providing evidence regarding which components are most promising in complex interventions. These findings should be considered in light of the risk of bias introduced by attrition to follow-up, which was high in this effectiveness trial with low barriers to participation.

**Trial Registration:**

International Standard Randomised Controlled Trial Number (ISRCTN) ISRCTN23310640; https://www.isrctn.com/ISRCTN23310640

**International Registered Report Identifier (IRRID):**

RR2-10.1136/bmjopen-2021-051044

## Introduction

Unhealthy behaviors, including alcohol consumption, unhealthy diets, physical inactivity, and smoking, are leading causes of noncommunicable diseases, including cardiovascular diseases, cancers, and diabetes [1-5]. The young adult years are a critical period for the establishment of healthy behaviors [6-8], which may reduce future morbidity [9-14]. Engaging in healthy behaviors also provides several immediate positive effects on both physical and mental health [15-20]. These include fewer mental health problems, such as anxiety and depression [17-20]; improved cognitive function and academic achievement [21,22]; and various aspects of well-being [15,16]. Additionally, reduced alcohol consumption lowers the risk of alcohol-related harms or negative consequences, such as social problems, injuries, or risky sexual behaviors [23,24]. However, a study of university students in 24 countries found a high prevalence of unhealthy behaviors [25]. In addition, unhealthy behaviors tend to co-occur among students; for instance, low fruit and vegetable consumption and low levels of physical activity often occur together. This is concerning because the risks of negative consequences associated with multiple unhealthy behaviors compound [26].

In the digital era, new avenues to support individuals in improving their health behaviors are emerging. One form of support is individual-level digital behavior change interventions, which deliver support materials via personal web portals, SMS text messages, smartphone apps, etc. The effectiveness of digital interventions across various health behaviors has been demonstrated in a wide range of populations and settings [27-33]. As young adults frequently use technology to access health information [34], digital interventions have the potential to efficiently reach this target group, and numerous single–health behavior digital interventions have been evaluated among university and college students [35-38]. In Sweden, digital interventions among university students have shown promising results, particularly with respect to alcohol [39-42], smoking cessation [43], and mental health promotion [44]. However, only a few interventions that support changes in multiple health behaviors simultaneously have been studied [45-47], and even fewer are tailored specifically for students [48]. Therefore, while single-behavior digital interventions show promise, our understanding of the effects of digital interventions targeting multiple health behaviors remains limited, despite the prevalence of co-occurring unhealthy behaviors [49,50].

Most digital behavior change interventions are grounded in behavior change theory and utilize behavior change techniques (BCTs) [51]. Systematic reviews [52,53] support the use of theory and BCTs in developing effective digital interventions. However, findings regarding the effectiveness of BCTs have been heterogeneous [54], indicating that the ways in which BCTs are implemented may vary considerably across interventions. In addition, behavioral interventions are typically evaluated using designs that estimate the effects of interventions as a whole [55], rather than at the component level, making it challenging to disentangle the contribution of specific components and BCTs to the overall effects.

In response to the lack of evidence on the effectiveness of digital interventions targeting multiple health behaviors, and the limited evidence on the effectiveness of different components of digital interventions, we conducted a factorial trial among university and college students in Sweden. The trial aimed to estimate the effectiveness of 6 intervention components of a digital intervention in promoting behavior change related to alcohol consumption, diet, physical activity, and smoking.

## Methods

### Study Design

We conducted a factorial randomized trial of a digital multiple health behavior intervention among university and college students in Sweden. The factorial trial was designed with 6 two-level factors representing the presence or absence of 6 intervention components. The treatment effect being estimated was thus the effect of the presence versus absence of each specific component, adjusted for the presence and absence of other components, as well as pairwise interactions between components. The trial was part of the MoBILE research program, which includes studies of digital health behavior interventions across the lifespan [56]. A protocol for the trial was published prospectively [57], and the trial was preregistered in the International Standard Randomised Controlled Trial Number (ISRCTN) registry on January 28, 2021 (ISRCTN23310640). This report follows CONSORT (Consolidated Standards of Reporting Trials) guidelines (see Multimedia Appendix 1) [58], including extensions of CONSORT for factorial trials [59].

Students were included in the trial if they met at least one of six inclusion criteria based on unhealthy behaviors as defined by national guidelines. We made an adjustment to the inclusion criteria for sugary drinks after trial registration. Initially, we decided that participants who consumed 3 or more units (33 cl) of sugary drinks per week would be included in follow-up analyses of sugary drink consumption. However, this was changed to 2-3 or more units per week because the questionnaire used did not differentiate between those consuming 2 or more units per week and those consuming 3 or more units per week. No other changes were made to the trial protocol.

### Study Setting, Recruitment, and Participants

Students at 18 geographically dispersed universities and colleges across Sweden (out of 50) were invited to participate in the trial. These institutions encompassed a variety of urban and rural settings and offered diverse educational profiles. Prospective participants were recruited using paper advertising (posters and leaflets), digital advertising (email, websites, and social media), and through student health care staff. Students registered their interest by sending an SMS text message to a dedicated phone number, after which they received an immediate reply with a hyperlink to a web page containing informed consent materials (see Multimedia Appendix 2). Consenting students were then asked to complete a baseline questionnaire (see Multimedia Appendix 3), the responses to which were used to assess eligibility. Included students were required to be 18 years or older and to fulfill at least one of the criteria presented in Textbox 1, which were based on Swedish national guidelines for healthy behaviors at the time the trial commenced.

Trial information and intervention materials were in Swedish and delivered to participants’ mobile phones; thus, individuals who did not understand Swedish well enough to enroll or who did not have access to a mobile phone were not able to take part in the study. Notably, it has been estimated that 96% of 16-35-year-olds in Sweden own a smartphone, and this percentage is likely to be even higher among university and college students.

**Criteria for inclusion.**

**1. Weekly alcohol consumption**: Consumed 10/15 (women/men) or more standard drinks of alcohol in the past week. A standard drink was defined as 12 g of pure alcohol.

**2. Heavy episodic drinking**: Consumed 4/5 (women/men) or more standard drinks of alcohol on a single occasion at least once in the past month.

**3. Fruit and vegetables**: Consumed less than 500 g of fruit and vegetables on average per day in the past week.

**4. Sugary drinks**: Consumed 2 or more units of sugary drinks in the past week. One sugary drink unit was defined as approximately 33 cl.

**5. Moderate-to-vigorous physical activity**: Spent less than 150 minutes on moderate-to-vigorous physical activity in the past week.

**6. Smoking**: Smoked at least one cigarette in the past week.

### Interventions

The digital multiple health behavior intervention studied in this trial was a mobile phone–based intervention targeting alcohol, diet, physical activity, and smoking. The intervention, called Buddy, consisted of 6 components aimed at promoting behavior change: screening and feedback (C1); goal-setting and planning (C2); motivation (C3); skills and know-how (C4); mindfulness (C5); and self-authored SMS text messages (C6). The components were informed by social-cognitive models of health [60] and have been included in interventions that have proven successful in facilitating behavior change [61,62]. Table 1 provides a brief description of each of the 6 components, utilizing the BCTTv1 93-item taxonomy [63] to specify BCTs. Please see Multimedia Appendix 4 for full details of the intervention.

**Table 1:** Description of intervention components, conceptual framework, practical application, and factorial conditions.

Component	Conceptual framework	Practical application	Factorial conditions
Component 1: Screening and feedback	*Self-monitoring* has been shown to be a potentially effective strategy for reducing excessive alcohol consumption [39,64-66] and promoting healthy eating and physical activity [67,68]. This concerned the following BCT^a^ components: Discrepancy between current behavior and goal(s) (BCT 1.6), Feedback on behavior (BCT 2.2), Self-monitoring of behavior (BCT 2.3), and Social comparison (BCT 6.2).	Every Sunday afternoon, participants received an SMS text message containing a hyperlink to a questionnaire regarding their current health behaviors. After completing the questionnaire, participants received feedback on their current behaviors in comparison with national guidelines. Thereafter, users were given access to the remaining components (depending on allocation).	When this component was absent, participants were not asked to complete the screening questionnaire but were instead shown the national guidelines without receiving personal feedback.
Component 2: Goal-setting and planning	*Self-regulatory* skills and capacity were addressed via goal-setting and planning. Planning-related activities, such as goal-setting, action planning, and practicing behavior, have been shown to be effective in lifestyle behavior interventions [67,69-74]. This concerned the following BCT components: Goal-setting (BCT 1.1), Problem solving (BCT 1.2), Action planning (BCT 1.4), Prompts/cues (BCT 7.1), and Behavior practice/rehearsal (BCT 8.1).	This component allowed participants to set 1 or more goals for their future behavior. It included action planning for how they would progress toward their goals, preparation for motivational struggles (coping planning), and strategies for rewarding themselves upon success. Participants could also create their own challenges or accept ready-made challenges for the coming week, such as walking for 15 minutes each day or not drinking any alcohol during the coming week. Reminders about their goals and challenges were sent to participants via SMS text messages throughout the week (up to 4 SMS text messages).	When absent, this component was not visible, and goal-setting reminders were not available.
Component 3: Motivation	Digital behavior change interventions have been shown to enhance *self-efficacy*; however, there is a lack of consensus across reviews regarding which content works best to facilitate increases in self-efficacy [75]. This concerned the following BCT components: Information about health consequences (BCT 5.1), Credible source (BCT 9.1), Pros and cons (BCT 9.2), and Comparative imagining of future outcomes (BCT 9.3).	This component contained information and tools to increase participants’ awareness of their own motivation, encourage commitment, and boost self-efficacy. It included information on negative health consequences, costs induced by certain behaviors, and reflective tasks delivered via SMS text messages, videos, and exercises. If participants chose, they could also activate motivational SMS text messages derived from previously developed and evaluated interventions [40,43,76-80], which were sent to them throughout the week (a maximum of 8-10 SMS text messages per week).	When absent, this component was not visible, and motivational SMS text messages were not available.
Component 4: Skills and know-how	Intervention content based on shaping knowledge, aimed at increasing an individual’s understanding and awareness to facilitate behavior change, has been shown to be effective [70,71,81,82]. This concerned the following BCT components: Instructions on how to perform a behavior (BCT 4.1), Behavior substitution (BCT 8.2), Habit formation (BCT 8.3), and Graded tasks (BCT 8.7).	This component aimed to increase participants’ skills and know-how by providing concrete tips on how to initiate and maintain lasting changes in everyday life through repetition and substitution. Participants were given strategies, for example, how to say no to alcoholic beverages or how to introduce vegetables into their meals. If participants chose to do so, they could also activate SMS text messages with tips and know-how, which were sent to them throughout the week (a maximum of 8-10 SMS text messages per week).	When absent, this component was not visible, and SMS text messages with tips and know-how were not available.
Component 5: Mindfulness	The mindfulness exercises were based on previous research and were considered evidence-based methods to improve the mental well-being of clinical populations, whereas their effects on behavior change in nonclinical settings are less well studied [83-87].	This component aimed to increase users’ awareness of their own lived experience and strengthen their capacity for a nonreactive, compassionate, and less stressful way of being in the world. Mindfulness exercises, including guided meditations, were offered to participants.	When absent, this component was not visible, and guided meditations were not available.
Component 6: Self-authored SMS text messages	SMS text message self-authorship is generally understudied in the literature, but was included in an effective digital alcohol intervention [88].	This component allowed participants to self-author up to 3 SMS text messages and schedule them to be sent to themselves throughout the week at times of their choosing. For example, a participant could write an SMS text message reminding themselves to eat 2 fruits each day, avoid drinking on Wednesdays, or go for a walk with a friend.	When absent, this component was not visible, and the ability to self-author SMS text messages was not available.

^a^BCT: behavior change technique.

**Figure 1:**
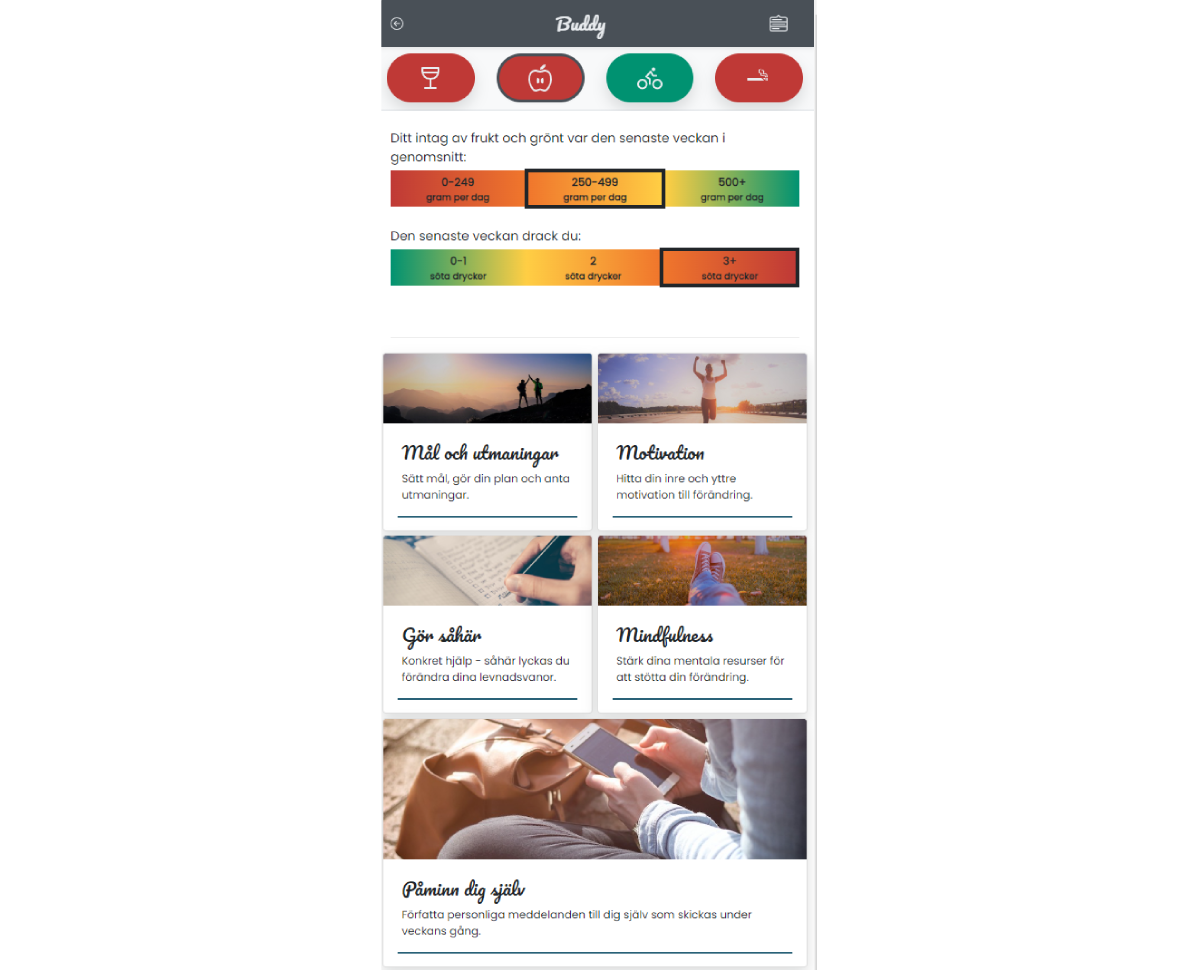
Screenshot of the Buddy intervention displaying the components in order from top to bottom and left to right: (1) Screening and feedback, (2) Goal-setting and planning, (3) Motivation, (4) Skills and know-how, (5) Mindfulness, and (6) Self-authored SMS text messages.

As shown in Figure 1, the components were presented to participants in a menu, allowing access in any order and with any frequency. Participants could therefore navigate between components in ways typical of smartphone apps. Regardless of the inclusion criterion met, all participants received intervention materials regarding alcohol, diet, physical activity, and smoking behaviors. There was no difference in the materials provided based on risk profile, except for the feedback received after screening (C1), where reported behavior was visually matched against national guidelines. The components presented materials that were both behavior-specific and more general to behavior change. Screening and feedback (C1) was conducted for each behavior, with feedback provided separately (eg, showing how current heavy drinking patterns match recommendations). Screening and feedback (C1) was offered to participants each week, allowing them to receive feedback on their behavior multiple times throughout the intervention period. Goal-setting and planning (C2) guided participants to set goals without prompting any specific behavior and also allowed them to create personal challenges; in addition, preprepared challenges were available and categorized by behavior. Motivation (C3) included materials that were not behavior specific and aimed to increase motivation and commitment to behavior change. The C3 component also allowed participants to sign up to receive behavior-specific SMS text messages throughout the week to boost motivation and commitment (participants could sign up for multiple behaviors). Skills and know-how (C4) contained content and optional SMS text messages designed to increase participants’ skills and knowledge about how to change, and was provided separately for each behavior, with loose references between behaviors (eg, recommending going for walks when feeling the urge to smoke). Mindfulness (C5) did not mention specific behaviors but instead aimed to strengthen the mental resources needed to change behavior. Self-authored SMS text messages (C6) provided a means for participants to send reminders to themselves throughout the week, with no content provided for any specific behavior.

Participants were randomly assigned to 1 of 64 conditions, each representing a unique combination of Buddy’s 6 components. The components assigned to individuals, based on their factorial allocation, were available for use at their discretion over a 4-month period, with weekly SMS text message reminders sent on Sunday afternoons to encourage access to the intervention.

### Outcomes and Measures

Outcomes are listed in Textbox 2 and described below. All questionnaires used in the trial can be found in Multimedia Appendix 3.

**Primary and secondary outcomes.**

**1. Primary outcomes**

- Alcohol: weekly alcohol consumption; monthly frequency of heavy episodic drinking.
- Diet: average daily consumption of fruit and vegetables; weekly consumption of sugary drinks.
- Physical activity: weekly moderate-to-vigorous physical activity.
- Smoking: 4-week point prevalence of smoking abstinence.

**2. Secondary outcomes**

- Weekly consumption of candy and snacks.
- BMI.
- Weekly number of cigarettes smoked.
- Perceived stress.

Weekly alcohol consumption was assessed by asking participants to report the number of standard drinks of alcohol they consumed in the past week (short-term recall method [89]). The frequency of heavy episodic drinking was assessed by asking participants how many times they consumed more than 4/5 (women/men) standard drinks of alcohol on 1 occasion in the past month. These 2 outcomes are both part of the core outcome set for brief alcohol interventions [90,91].

Diet and physical activity were assessed using a questionnaire based on a previously published questionnaire by the National Board of Health and Welfare in Sweden [92], which was further modified to include portion sizes. Fruit and vegetable consumption was assessed using 2 questions on the number of portions (100 g) of fruit and vegetables, respectively, that participants consumed on average per day during the past week. Sugary drink consumption was assessed using a question on the number of units (33 cl) of sugary drinks participants consumed in the past week. Moderate-to-vigorous physical activity (MVPA) was assessed by summing responses to 2 questions on the number of minutes spent on MVPA, respectively, during the past week.

BMI was calculated using participants’ self-reported weight (without further instructions). Baseline height was used, as it was deemed unlikely to have changed significantly.

Four-week point prevalence of smoking abstinence (no cigarettes in the past 4 weeks) was assessed as a binary question, a measure suggested by the Society for Research on Nicotine and Tobacco [93]. Participants who reported having smoked any cigarettes in the past 4 weeks were also asked to report the number of cigarettes smoked in the past week.

Perceived stress was assessed using the Short-Form Perceived Stress Scale [94].

### Follow-Up

There were 2 follow-up intervals for primary and secondary outcomes: 2 and 4 months after randomization. The 2-month follow-up facilitated the assessment of the immediate effects of the components, while the 4-month follow-up facilitated estimation of effects after prolonged access. Additional longer follow-up intervals were not included to reduce participant burden and because attrition was expected to be much higher at longer intervals from baseline. An additional follow-up was conducted at 1 month after randomization to assess proposed mediators of the intervention; these results will be reported separately. All follow-ups were initiated by sending SMS text messages to participants with hyperlinks to questionnaires (see Multimedia Appendix 3). Additional attempts were made to collect data from nonresponders, including 2 SMS text message reminders sent 2 days apart, followed by phone calls with a maximum of 5 attempts.

### Randomization and Blinding

We used block randomization with random block sizes of 64 and 128 to allocate participants equally among conditions, where each condition represented a unique combination of components. The process was automated, with the sequence computer-generated and the backend server allocating eligible participants after completion of the baseline. Neither research personnel nor participants were able to influence the allocation. Research personnel were blind to allocation throughout the trial. Participants were not aware of components other than those to which they were allocated and were therefore considered blind to allocation.

### Statistical Analysis

We conducted analyses with participants in the conditions to which they were randomly allocated (intention-to-treat). We estimated models using both available data and data with missing values imputed. We used multiple imputation with chained equations, generating 100 datasets using 30 iterations with predictive mean matching. To account for the uncertainty inherent in missing data, imputed analyses were performed for all models as a sensitivity analysis, regardless of the proportion of missingness. All statistical analyses were prespecified in the trial protocol [57].

Longitudinal data were modeled using multilevel models with random intercepts for participants and time-by-component interactions. In addition to estimating the marginal effects of each component, we estimated the effects of pairwise interactions among components to examine potential synergistic effects. This was done by including pairwise interaction terms in the regression models for all outcomes. The treatment effect being estimated was thus the effect of the presence versus absence of each specific component, adjusted for the presence and absence of other components, as well as pairwise interactions between components. The models were adjusted for age, gender, and baseline measures of importance, confidence, and knowledge of how to change.

Bayesian inference was used to estimate the parameters of the models, using standard normal priors for coefficients to incorporate an a priori conservative view of the magnitudes of effects [95]. The standard normal priors induced shrinkage in the parameter estimates to protect against spurious findings. We report the marginal posterior probability of effect (POE), with the median of the posterior distribution representing a point estimate of the magnitude of the effect, along with 95% compatibility intervals (CoIs) defined by the 2.5th and 97.5th percentiles.

### Primary and Secondary Outcomes

We analyzed primary outcomes at 2 and 4 months among participants who fulfilled the respective inclusion criteria. For example, the MVPA outcome was analyzed among those who reported less than 150 minutes of MVPA in the past week at baseline. Secondary outcomes at 2 and 4 months were analyzed among all participants, except for the number of cigarettes smoked in the past week, which was analyzed among baseline smokers who continued to smoke.

Weekly alcohol consumption, frequency of heavy episodic drinking per month, number of sugary drinks per week, weekly intake of candy and snacks, and cigarettes smoked in the past week were all analyzed using negative binomial regression. Average intake of fruit and vegetables per day, MVPA minutes per week, BMI, and stress were all analyzed using linear regression. Point prevalence of smoking abstinence was analyzed using logistic regression.

### Ancillary Analyses

We investigated attrition using 2 approaches. First, we estimated the odds of responding to follow-up, conditional on baseline characteristics and the presence or absence of the 6 components (factors), using logistic regression. One model included no interaction terms, while a second model included interaction terms between factors and baseline characteristics. Given the large number of covariates in these models, we used Cauchy priors to promote a parsimonious model (centered at 0 with a standard normal hyperprior for the scale). The use of Cauchy priors encodes an a priori conservative view and pulls estimated associations toward the null unless the data strongly suggest otherwise. Second, if data are missing systematically, nonresponders may be more similar to responders who require more attempts to collect data than to those who require fewer attempts. We therefore modeled primary outcome data conditional on the number of attempts required to collect follow-up data. Associations between the number of attempts and outcome measures may indicate systematic attrition.

### Post Hoc Analyses

To investigate whether the number of components that participants had access to affected outcomes, we conducted post hoc analyses in which outcomes were modeled conditional on the number of components. The same model specifications were used as in the primary analyses, with dummy variables for components replaced by a variable representing the component count.

### Sample Size

We used a Bayesian sequential design to monitor when to stop recruitment [96]. In such designs, targets are defined and evaluated throughout the course of the trial to determine whether the trial should stop or continue. As 4-month follow-up data became available, we modeled each of the primary outcomes according to the analysis plan, and coefficients for dummy variables representing the presence or absence of each component were compared against targets for effect, harm, and futility (see protocol for details of the criteria [57]).

We stopped recruitment after 30 months. While our prespecified limit was 24 months of recruitment irrespective of targets, we extended recruitment by 6 months because some colleges and universities began their recruitment procedures later than others. Overall, each participating college and university was expected to recruit for at least 24 months.

### Ethical Considerations

The trial received ethical approval from the Swedish Ethical Review Authority on December 15, 2020 (Dnr: 2020-05496 and Dnr: 2022-03561-02). All participants were provided with study information and gave informed consent before responding to the baseline questionnaire and subsequently being randomized. Participants received no compensation for participating in the trial. Participants’ phone numbers were encrypted and stored in the study database to link baseline and follow-up data. At project completion, the encrypted phone numbers will be deleted, and the data will be anonymized.

## Results

### Overview

**Figure 2:**
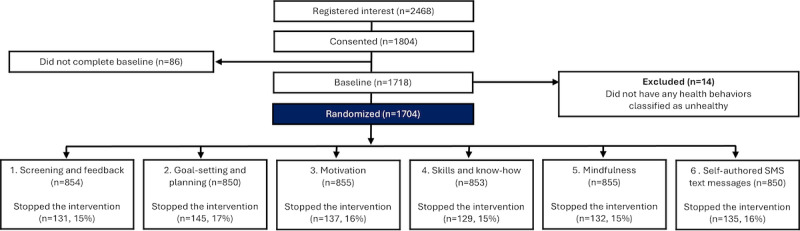
CONSORT (Consolidated Standards of Reporting Trials) participant flow diagram—sign up and randomization.

**Figure 3:**
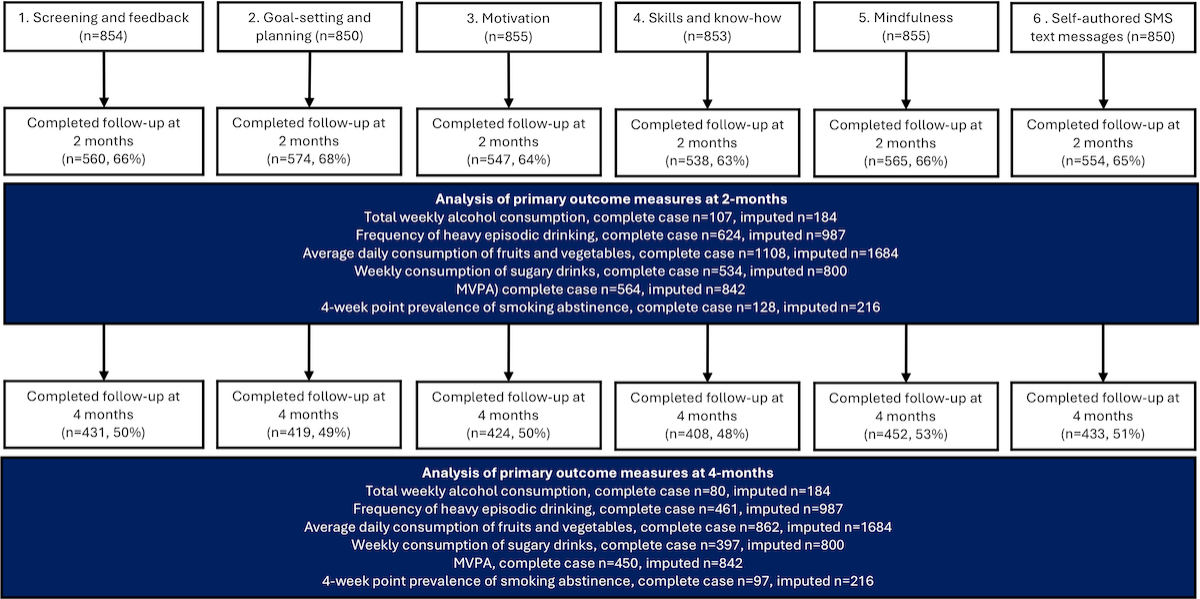
CONSORT (Consolidated Standards of Reporting Trials) participant flow diagram—follow-up.

A CONSORT participant flow diagram is presented in Figure 2 (sign up and randomization) and Figure 3 (follow-up). Between April 13, 2021, and October 18, 2023, 2468 individuals expressed interest in the study, of whom 1804 consented. The majority were recruited through the trial’s information materials, either digital or leaflets provided by university student health care services (1999/2468, 81%), while the remainder were recruited through online advertisements on social media platforms (469/2468, 19%). A total of 1718 individuals completed the baseline questionnaire, of whom 14 were excluded for not fulfilling any of the behavioral inclusion criteria. The remaining 1704 participants were randomized. Baseline characteristics of the randomized participants were balanced across all factors (see Table 2).

**Table 2:** Baseline characteristics of randomized participants.

Characteristics		Total (n=1704)	Screening and feedback (n=854)	Goal-setting and planning (n=850)	Motivation (n=855)	Skills and know-how (n=853)	Mindfulness (n=855)	Self-authored SMS text messages (n=850)
**Sex, n (%)**								
	Women	1400 (82.16)	709 (83.02)	704 (82.82)	699 (81.75)	718 (84.17)	697 (81.52)	692 (81.41)
	Men	304 (17.84)	145 (16.98)	146 (17.18)	156 (18.25)	135 (15.83)	158 (18.48)	158 (18.59)
Age (years), mean (SD)		28 (6.9)	28.3 (6.9)	28.5 (7.1)	28.2 (7.0)	27.8 (6.8)	28.5 (7.0)	28.4 (7.0)
**BMI (kg/m^2^), mean (SD)**		25.7 (5.5)	25.9 (5.5)	25.6 (5.3)	25.7 (5.2)	25.8 (5.6)	25.7 (5.3)	25.8 (5.3)
	Underweight (<18.5), n (%)	60 (3.52)	28 (3.28)	33 (3.88)	30 (3.51)	33 (3.87)	27 (3.16)	19 (2.24)
	Normal weight (18.5-24.99), n (%)	844 (49.53)	417 (48.83)	413 (48.59)	415 (48.54)	429 (50.29)	426 (49.82)	432 (50.82)
	Overweight (25-29.99), n (%)	458 (26.88)	234 (27.40)	236 (27.76)	239 (27.95)	214 (25.09)	241 (28.19)	227 (26.71)
	Obese (≥30.0), n (%)	342 (20.07)	175 (20.49)	168 (19.76)	171 (20.00)	177 (20.75)	161 (18.83)	172 (20.24)
**Alcohol, mean (SD)**								
	Total weekly alcohol consumption	3.7 (5.2)	3.7 (5.2)	3.8 (5.3)	3.7 (5.1)	3.6 (5.1)	3.8 (5.1)	3.7 (5.1)
	Frequency of heavy episodic drinking	1.8 (2.7)	1.8 (2.7)	1.8 (2.8)	1.8 (2.7)	1.8 (2.7)	1.7 (2.4)	1.8 (2.5)
**Dietary behavior, mean (SD)**								
	Average daily fruit and vegetable consumption, portions per week	1.3 (1.1)	1.3 (1.1)	1.3 (1.1)	1.3 (1.1)	1.3 (1.1)	1.3 (1.1)	1.3 (1.1)
	Sugary drinks, cans per week	2.7 (4.1)	2.7 (4.1)	2.7 (4.2)	2.7 (4.1)	2.6 (3.9)	2.7 (4.0)	2.5 (3.9)
	Candy and snacks, portions per week	6.5 (6.4)	6.6 (6.4)	6.4 (6.3)	6.6 (6.5)	6.5 (6.4)	6.5 (6.5)	6.3 (6.4)
**Physical activity, mean (SD)**								
	MVPA (minutes)	207 (214)	209 (221)	214 (225)	213 (214)	211 (213)	207 (210)	196 (207)
**Smoking**								
	Number of smokers, n (%)	218 (12.79)	106 (12.41)	119 (14.00)	106 (12.40)	105 (12.31)	104 (12.16)	101 (11.88)
	Number of cigarettes last week, mean (SD)	3.5 (16.6)	3.0 (15.1)	4.2 (18.8)	3.6 (17.7)	2.6 (13.4)	3.3 (16.5)	2.8 (14.9)
**Stress, mean (SD)**								
	Self-perceived stressª	7.8 (2.9)	7.8 (2.9)	7.8 (2.9)	7.7 (3.0)	7.8 (2.9)	7.7 (3.0)	7.7 (2.9)
**Psychosocial measures, median (quartiles)**								
	Importance^b^	8 (7-10)	8 (7-10)	8 (7-10)	8 (7-10)	8 (7-10)	8 (7-10)	8 (7-10)
	Confidence^b^	6 (5-8)	6 (5-8)	6 (5-8)	6 (5-8)	6 (5-8)	6 (5-8)	6 (5-8)
	Know-how^b^	6 (5-8)	6 (5-8)	6 (5-8)	6 (5-8)	6 (5-8)	6 (5-8)	6 (5-8)

^a^The 4-item Perceived Stress Scale with total scores ranging from 0 to 16.

^b^Single item with 1-10 response options.

Among participants, 16% stopped the intervention before the 16-week program had ended. Consistent with the consent provided, we did not investigate the reasons why these individuals chose to stop receiving SMS text messages. The CONSORT flowchart in Figure 2 provides the numbers who stopped per component. All participants, including those who discontinued the intervention, were followed up and included in the main analysis. At the 2-month follow-up interval, primary outcome data were available for 1118 out of 1704 (65.61%) participants, and for 874 out of 1704 (51.29%) participants at the 4-month follow-up interval.

### Attrition

Attrition analyses revealed no marked associations between baseline characteristics and nonresponse at the 2-month follow-up interval. At the 4-month follow-up interval, there was evidence that older participants were more likely to respond to follow-up and that those smoking more cigarettes per week at baseline were less likely to respond. Notably, the association between nonresponse and smoking was driven by a few data points. Neither of these associations was found to differ between component conditions. Please see Multimedia Appendix 5 for full details.

There was some evidence that late responders, that is, those requiring more attempts to collect follow-up data, had healthier behaviors regarding fruit and vegetable intake and alcohol consumption. This association was attenuated among those with access to the screening and feedback (C1) component, who consumed more alcohol and sugary drinks at the 4-month follow-up and less fruit and vegetables at the 2-month follow-up. While there is considerable uncertainty surrounding these analyses, under the assumption that late responders are more similar to nonresponders than early responders, this may indicate systematic attrition. Please see Multimedia Appendix 6 for details.

### Primary and Secondary Outcomes

#### Estimates and Main Effects

Table 3 contains the main effects of individual components on primary outcomes, and Multimedia Appendix 7 presents the main effects of individual components and 2-way interactions between components for the available data. The corresponding analyses with missing data imputed are presented in Multimedia Appendix 8. Here, we highlight findings for which the evidence of effects was strongest. We report estimates from analyses of the available data unless otherwise noted.

**Table 3:** Estimates of marginal effects of components on primary outcomes at 2- and 4-month follow-up.

Component								C1					C2					C3					C4					C5					C6		
								Estimate^a^		Probability^b^, %		Estimate			Probability, %		Estimate			Probability, %		Estimate			Probability, %		Estimate			Probability, %		Estimate			Probability, %
**Weekly alcohol consumption**																																			
						2 months		1.06 (0.77 to 1.50)		63.7		0.95 (0.68 to 1.32)			63.1		0.79 (0.58 to 1.08)			93.6		0.91 (0.66 to 1.25)			71.9		0.98 (0.72 to 1.34)			53.9		0.90 (0.65 to 1.23)			75.5
						4 months		1.01 (0.71 to 1.45)		53.1		1.06 (0.74 to 1.53)			63.4		0.87 (0.62 to 1.22)			79.6		0.96 (0.67 to 1.36)			59.9		1.19 (0.84 to 1.69)			83.4		0.65 (0.45 to 0.93)			99.1
**Heavy episodic drinking**																																			
					2 months		0.87 (0.74 to 1.02)		95.2		0.89 (0.75 to 1.04)			93.1		0.96 (0.82 to 1.13)			68.7		1.00 (0.86 to 1.18)			51.7		0.89 (0.76 to 1.04)			92.5		1.14 (0.98 to 1.34)			95.0	
					4 months		0.97 (0.80 to 1.16)		65.2		1.09 (0.91 to 1.32)			82.8		1.18 (0.99 to 1.42)			96.5		1.03 (0.86 to 1.25)			64.2		1.11 (0.92 to 1.33)			86.1		0.93 (0.77 to 1.12)			78.6	
**Fruit and vegetable consumption**																																			
				2 months				0.11 (–0.02 to 0.24)		94.7		–0.09 (–0.22 to 0.04)			90.2		0.09 (–0.04 to 0.23)			92.0		0.19 (0.06 to 0.33)			99.8		0.03 (–0.11 to 0.16)			64.7		–0.01 (–0.14 to 0.12)			54.9
				4 months				0.12 (–0.03 to 0.26)		94.4		–0.01(–0.15 to 0.14)			53.6		0.12 (–0.02 to 0.27)			95.4		0.14 (–0.01 to 0.28)			96.9		–0.05 (–0.19 to 0.09)			74.9		–0.09 (–0.23 to 0.05)			88.2
**Sugary drinks**																																			
			2 months					0.91 (0.77 to 1.06)		88.4		1.03 (0.87 to 1.21)			61.2		0.98 (0.84 to 1.15)			59.2		1.04 (0.88 to 1.22)			66.5		0.94 (0.80 to 1.10)			78.8		1.24 (1.05 to 1.46)			99.4
			4 months					1.03 (0.86 to 1.24)		62.2		1.05 (0.87 to 1.26)			68.8		1.09 (0.90 to 1.31)			81.5		1.06 (0.88 to 1.27)			74.2		1.07 (0.89 to 1.29)			76.6		1.16 (0.96 to 1.39)			93.8
**Moderate-to-vigorous physical activity**																																			
		2 months						9.34 (–25.6 to 45.5)		69.7		–5.73 (–41.9 to 29.5)			63.0		35.8 (–0.41 to 71.9)			97.4		17.1 (–19.2 to 54.2)			82.2		41.2 (5.04 to 77.6)			98.7		5.68 (–30.5 to 41.1)			62.2
		4 months						28.1 (–11.3 to 67.6)		92.0		17.6 (–20.6 to 56.5)			81.4		6.63 (–33.1 to 45.2)			63.0		32.8 (–6.1 to 71.8)			95.2		3.02 (–36.3 to 42.0)			56.0		–19.2 (–57.6 to 20.1)			83.6
**Smoking cessation**																																			
	2 months						1.12 (0.35 to 3.59)		57.4		0.56 (0.17 to 1.76)			84.3		1.43 (0.43 to 4.63)			72.3		0.68 (0.21 to 2.23)			74.5		0.46 (0.14 to 1.49)			90.4		2.15 (0.67 to 6.74)			90.4	
	4 months						1.45 (0.35 to 6.10)		69.2		0.48 (0.11 to 2.00)			84.3		2.30 (0.52 to 9.56)			87.3		0.77 (0.17 to 3.39)			64.0		0.55 (0.13 to 2.28)			79.6		0.94 (0.22 to 4.02)			53.0	

^a^Estimates are presented as the median of the posterior distribution with 95% compatibility intervals. Incidence rate ratios are presented for weekly alcohol consumption, heavy episodic drinking, sugary drinks; odds ratios are presented for smoking cessation; and mean differences are presented for fruit and vegetable consumption and moderate-to-vigorous physical activity.

^b^Probability is the proportion of the posterior distribution above or below the null in the direction of the median.

#### Primary Outcomes

##### Total Weekly Alcohol Consumption

Self-authored SMS text messages (C6) reduced total weekly alcohol consumption at 4 months (incidence rate ratio [IRR] 0.65, CoI 0.45-0.93; POE 99.1%). Apart from weak evidence for an effect of motivation (C3) at 2 months, there was no marked evidence of effects for the other components individually. There was evidence that combining motivation (C3) and self-authored SMS text messages (C6) had a larger effect on alcohol consumption at 4 months (IRR 0.53, CoI 0.31-0.88; POE 99.4%). The evidence for the combined effect was also marked in the imputed analyses. Notably, relatively few participants drank above national guidelines with respect to weekly alcohol consumption, and therefore, the numbers included in these analyses were smaller than for other behaviors (see Figure 3 and Multimedia Appendices 7 and 8).

##### Frequency of Heavy Episodic Drinking

There was considerable variability in the evidence for the effectiveness of components on heavy episodic drinking, with evidence suggesting both benefit and harm. Screening and feedback (C1) reduced the frequency of heavy episodic drinking at 2 months (IRR 0.87, CoI 0.74-1.02; POE 95.2%), which was also evident in the imputed analyses. However, the effect was not sustained at the 4-month follow-up. The strongest evidence for benefit was observed for the combination of screening and feedback (C1) with goal-setting and planning (C2) (IRR 0.78, CoI 0.62-0.98; POE 98.4%), and screening and feedback (C1) with mindfulness (C5) (IRR 0.78, CoI 0.62-0.98; POE 98.4%) at 2 months. Similar evidence was found for the combination of goal-setting and planning (C2) with mindfulness (C5). Evidence of harm was most prominent for motivation (C3), both alone and in combination with goal-setting and planning (C2) or mindfulness (C5) (IRR 1.28, CoI 0.99-1.64; POE 97.1%) at the 4-month follow-up interval. These harmful effects were not evident at the 2-month interval. Notably, the evidence of harm was less prominent in the imputed analyses, whereas the evidence for benefit remained.

##### Daily Portions of Fruit and Vegetables

The strongest evidence for the beneficial effects of single components on fruit and vegetable consumption was found for screening and feedback (C1) and skills and know-how (C4) at both follow-up intervals. The largest effect was observed for the combination of these 2 components at 2 months (mean difference 0.30, CoI 0.11-0.48; POE 99.9%) and at 4 months (mean difference 0.26, CoI 0.05-0.46; POE 99.4%). While the evidence for motivation (C3) alone was not convincingly strong, in combination with screening and feedback (C1) or skills and know-how (C4), there was evidence of an effect at both the 2- and 4-month intervals on par with the effect observed for the combination of screening and feedback (C1) and skills and know-how (C4). These findings all persisted in the imputed analyses.

##### Weekly Consumption of Sugary Drinks

There was no strong evidence of the beneficial effects of the intervention components on sugary drink consumption. Rather, there was strong evidence of harm from self-authored SMS text messages (C6) at the 2- and 4-month intervals. The evidence suggested an increase of 15%-20% in weekly consumption of sugary drinks from this component alone, and the effect was larger in combination with goal-setting and planning (C2), motivation (C3), and skills and know-how (C4). These negative effects were partially sustained in the imputed analyses.

##### Weekly Moderate-to-Vigorous Physical Activity

There was evidence of beneficial effects on MVPA from several combinations of components at the 2-month interval; however, few of these effects persisted at the 4-month interval. The evidence for an effect of single components was strongest for motivation (C3), which increased weekly minutes of MVPA at 2 months (mean difference 35.8, CoI −0.41 to 71.9; POE 97.4%), and mindfulness (C5), which also increased minutes of MVPA at 2 months (mean difference 41.2, CoI 5.04-77.6; POE 98.7%). When motivation (C3) and mindfulness (C5) were combined, the effect on minutes of MVPA was even greater at 2 months (mean difference 78.0, CoI 28.3-128.2; POE 99.9%). Combining motivation (C3) with skills and know-how (C4) also had a positive effect on MVPA at 2 months. The imputed analyses produced similar findings.

While there was only weak evidence that screening and feedback (C1) alone affected MVPA, there was evidence that combining screening and feedback (C1) with skills and know-how (C4) increased minutes of MVPA at 4 months (mean difference 60.1, CoI 3.6-116.5; POE 98.2%), although the evidence was not as strong in the imputed analyses. In addition, there was evidence that screening and feedback (C1) combined with mindfulness (C5) increased minutes of MVPA at the 2-month interval (mean difference 50.6, CoI −0.55 to 101.9; POE 97.4%), with similar findings in the imputed analyses.

##### Smoking

There was weak evidence of both benefit and harm from the intervention components, and their combinations, on smoking cessation. The relatively strongest evidence for benefit was found for self-authored SMS text messages (C6) at 2 months (odds ratio 2.15, CoI 0.67-6.74; POE 90.4%), and for the combination of screening and feedback (C1) and motivation (C3) at 4 months (odds ratio 4.28, CoI 0.49-39.9; POE 90.7%). The strongest evidence for harm was found for the combination of goal-setting and planning (C2) and mindfulness (C5) at 2 months (odds ratio 0.23, CoI 0.04-1.27; POE 95.4%). The evidence for both benefit and harm persisted in the imputed analyses, although effect estimates were attenuated toward the null.

#### Secondary Outcomes

##### Weekly Portions of Candy and Snacks

At the individual component level, there was some evidence that motivation (C3) decreased candy and snack consumption at both 2 and 4 months. When motivation (C3) was combined with screening and feedback (C1), the strongest evidence for benefit was observed at 4 months (IRR 0.85, CoI 0.71-1.01; POE 96.8%). Increases in candy and snack consumption were observed for mindfulness (C5) alone at 4 months and self-authored SMS text messages (C6) alone at 2 months. While the evidence for these effects was not strong, it persisted in the imputed analyses. In addition, combining mindfulness (C5) and self-authored SMS text messages (C6) increased candy and snack consumption at 4 months (IRR 1.21, CoI 1.01-1.44; POE 97.7%). These findings also persisted in the imputed analyses.

##### BMI

At the 2-month interval, there was evidence that mindfulness (C5) and self-authored SMS text messages (C6) increased BMI, both individually and when combined (combined mean difference 0.34, CoI 0.09-0.59; POE 99.6%). However, evidence for these effects was not observed at the 4-month interval. There was also evidence that combining screening and feedback (C1) with mindfulness (C5) resulted in higher BMI at both the 2- and 4-month intervals. These findings persisted in the imputed analyses.

##### Cigarettes Smoked Per Week

Regarding the number of cigarettes smoked per week among those who continued to smoke, there was evidence that the combination of screening and feedback (C1) and goal-setting and planning (C2) had a beneficial effect at 2 months, and that combining motivation (C3) and self-authored SMS text messages (C6) had a harmful effect at 2 months.

##### Perceived Stress

There was evidence of a beneficial effect on stress from the skills and know-how (C4) component alone at 4 months (mean difference −0.35, CoI −0.71 to 0.01; POE 97.1%). The effect size increased when skills and know-how (C4) was combined with screening and feedback (C1) or motivation (C3). These findings were not markedly different in the imputed analyses.

##### Post-Hoc Analyses

In Multimedia Appendix 9, we present estimates of the effects of the number of components participants had access to on primary and secondary outcomes. The relatively strongest evidence was found for fruit and vegetable consumption at 2 months (0.05 portion increase per module, CoI 0.001-0.11; POE 97.6%) and 4 months (0.04 portions per module, CoI −0.02 to 0.1; POE 90.7%), as well as for weekly MVPA at the 2-month follow-up (17.5 minutes per module, CoI 2.9-32.2; POE 99.0%) and 4 months (11.8 minutes per module, CoI −3.8 to 27.1; POE 93.1%).

### Intervention Access

Of the 854 participants who had access to the screening and feedback component (C1), 807 (94.5%) screened themselves at least once during the intervention period. Among those who screened themselves at least once, 356 (44.1%) did so 4 or more times, while 129 (16.0%) screened themselves 3 times, 161 (20.0%) 2 times, and 161 (20.0%) 1 time.

Of the 850 participants who had access to the goal-setting and planning component (C2), 650 (76.5%) accessed the component at least once. Among all participants, 427 (50.2%) set at least one goal, and 403 (47.4%) selected or self-authored at least one challenge using the intervention’s technical solution.

Of the 850 participants who had access to the motivation component (C3), 554 (65.2%) accessed the content at least once. Among all participants, 169 (19.9%) activated SMS text messages for physical activity, 166 (19.5%) for diet, 47 (5.5%) for alcohol, and 21 (2.5%) for smoking. The motivation component provided interactive content on motives, financial costs, and health consequences, with 294 recorded engagements using the intervention’s technical solution.

Of the 855 participants who had access to the skills and know-how component (C4), 536 (62.7%) accessed the materials at least once. Among all participants, 166 (19.4%) activated SMS text messages for physical activity, 172 (20.1%) for diet, 51 (6.0%) for alcohol, and 28 (3.3%) for smoking. Most of the content in this module consisted of text and visual materials designed to build knowledge and skills for behavior change. A small number of interactive exercises were also included, with 283 recorded engagements using the intervention’s technical solution.

Of the 855 participants who had access to the mindfulness component (C5), 435 (50.9%) accessed the component at least once. Meditations were accessed by 87 (10.2%) participants, and 156 (18.2%) completed at least one task. Among those who completed tasks, 76 (48.7%) completed 3 or more tasks, 33 (21.2%) completed 2 tasks, and 47 (30.1%) completed 1 task. Some tasks required participants to write longer texts, which could be completed either using the intervention’s technical solution or offline; however, only activities completed using the technical solution could be measured.

Of the 850 participants who had access to the self-composed SMS text messages component (C6), 406 (47.8%) accessed the component at least once. Among those who accessed the component, a total of 570 SMS text messages were composed by 176 participants.

We had no means of accurately measuring the time spent on each component and therefore, cannot approximate the time spent reading the text and visual information provided in the components. Additionally, we had no means of measuring the extent to which participants comprehended and adopted the information provided. For instance, the skills and know-how component (C4) included mostly tips and information on concrete actions for participants to take, and we had no means of measuring how much time participants spent reading and engaging with the support provided.

## Discussion

### Principal Findings

This factorial randomized trial estimated the effectiveness of 6 behavior change components within a digital multiple health behavior intervention. We found evidence that both individual components and 2-way interactions between components led not only to notable improvements in health behaviors, but also to notable harm. The components screening/feedback (C1), motivation (C3), and skills/know-how (C4) each demonstrated individual and interacting improvements across various behavioral outcomes, including reductions in alcohol consumption, smoking, and stress, as well as increases in fruit and vegetable consumption and physical activity. Most effects were observed at 4 months; however, changes in heavy episodic drinking and physical activity were noted only at the 2-month follow-up interval.

Previous factorial trials of health behavior change interventions have shown mixed results regarding the effectiveness of individual components. In general, combining components tends to lead to more substantial improvements in health behaviors compared with individual components alone. For instance, Crane et al [97] found that although individual modules of the Drink Less app did not affect weekly alcohol consumption or Alcohol Use Disorders Identification Test scores, 2-way interactions between components did. Specifically, the combination of normative feedback and cognitive bias retraining reduced weekly alcohol consumption, whereas self-monitoring, feedback, and action planning improved Alcohol Use Disorders Identification Test scores. Similarly, in our study, screening/feedback (C1) reduced heavy episodic drinking at 2 months, with enhanced effects when combined with goal-setting/planning (C2) or mindfulness (C5). Notably, the study by Crane et al [97] was not restricted to university students and focused solely on alcohol consumption.

Schroé et al [98] found that the MyPlan2.0 digital intervention increased MVPA and reduced sedentary behavior through self-monitoring. Additionally, coping planning further boosted MVPA, with the combination of action planning, coping planning, and self-monitoring proving most effective at 5 weeks. Similarly, Fanning et al [99] evaluated a 12-week physical activity app incorporating self-monitoring, goal-setting, and feedback, resulting in an 11-minute increase in daily MVPA (from 34 to 46 minutes). Participants who received points-based feedback engaged in an additional 6 minutes of MVPA per day. Our findings indicated that combining components such as screening/feedback (C1), motivation (C3), skills/know-how (C4), and mindfulness (C5) enhanced short-term physical activity. However, this increase was not sustained over time, suggesting that although the components were effective at 2 months, they did not support the longer-term maintenance of increased physical activity at 4 months. The dissimilarities in findings may be due to both Schroé et al [98] and Fanning et al [99] focusing exclusively on physical activity and not restricting their samples to university students.

The evidence showed no effect for the goal-setting/planning component (C2) when delivered alone. Harms were observed for the interaction between goal-setting/planning (C2) and mindfulness (C5) on smoking cessation at 2 months. Additionally, self-authored SMS text messages (C6) increased sugary drink consumption, with the effect amplified when combined with goal-setting/planning (C2), motivation (C3), or skills/know-how (C4). These findings regarding sugary drinks may be related to the observed decrease in alcohol consumption, as individuals might substitute sugary drinks for alcohol, and behavior substitution was part of the advice provided to participants. Additionally, students may consume energy drinks during studying and physical activity, further contributing to increased sugary beverage intake. Previous research [100] highlighted the complexity of young adults’ perceptions of beverage healthfulness, which are influenced by factors including sugar content, packaging, marketing, and context of consumption. For example, a drink may be perceived as healthier if consumed during physical activity or as part of a balanced meal.

Although beneficial, health behavior interventions can increase stress and health anxiety due to heightened health consciousness, dissonance with desired behaviors, or pressure to meet goals, all of which may negatively affect mental well-being [101]. However, our study found that perceived psychological stress decreased at both the 2- and 4-month intervals when participants were provided with the skills/know-how component (C4). This effect became more pronounced over time, highlighting the importance of equipping individuals with practical skills and knowledge to manage their health in daily life.

Taking a broader perspective beyond the scope of factorial trials, our findings differ from previous digital multiple health interventions for young adults and university students, which have reported small effect sizes when evaluating interventions as a whole. For instance, Champion et al [102] reported that a digital multiple health intervention showed no evidence of effect in modifying lifestyle behaviors among adolescents in Australia; however, this study included a much younger demographic (ages 11-13) than our study. The online intervention U@Uni by Cameron et al [103], which targeted new university students, found no evidence of effect on the 4 primary outcomes: portions of fruit and vegetables per day, physical activity in the previous week, alcohol consumption in the previous week, and smoking status at the 6-month follow-up. However, U@Uni also targeted a younger demographic of incoming university students who may not have had the same motivation to change as students who had already established themselves. By contrast, similar to our study, Duan et al [104] demonstrated positive effects on physical activity and fruit and vegetable consumption after 8 weeks of a sequentially delivered web-based intervention among Chinese college students.

### Generalizability and Limitations

This trial employed a pragmatic recruitment strategy, reaching students at universities across Sweden through channels typically used by Student Health Care Centers to provide health information and services. This open invitation, with broad inclusion criteria and low barriers to participation, targeted students with at least one health behavior categorized as a significant risk factor for their physical or mental health. Although the validity of trials is not contingent on the sample being representative of the target population, in our case, we anticipate that those who would use the intervention in a real-world rollout would be similar to those who participated in this trial. Therefore, we argue that this trial estimates the effectiveness, rather than the efficacy, of the intervention components. Relatedly, we do not have data to determine which recruitment source led to each individual’s participation; thus, a limitation of the study design is that we cannot assess whether effectiveness was moderated by mode of recruitment.

In line with our aim to estimate effectiveness, we designed the study to gain knowledge about how access to components affects outcomes in a large population, rather than to examine the efficacy of different components in controlled environments. Effectiveness, as a concept, seeks to understand what happens under real-world conditions; for this reason, we neither expected nor observed that all participants would use all of the intervention materials available to them. Nevertheless, in this study, which proactively recruited university students, 768 of 854 (89.9%) with access to the screening component used it at least once, and approximately one-half of participants with access to the other components used them at least once. Based on the measures available to us, the 2 components with the lowest usage were mindfulness (C5) and self-authored SMS text messages (C6). This is not surprising, as both require a stronger cognitive commitment, and mindfulness was not advertised to participants before enrollment; thus, there may have been limited capacity or interest to engage with these components. However, even if a smaller proportion of individuals engage with specific components, it may still be beneficial to include them in interventions, as they may have positive effects from a population perspective. Overall, the findings from this study should be understood as generalizing to the proactive dissemination of digital interventions rather than as controlled treatment of specific individuals.

The invitation reached individuals who were not necessarily actively seeking help but who could benefit from it and were willing to participate when approached. Although recruitment did not target students who had explicitly requested support for change, baseline measures indicated a gap between participants’ ratings of importance and their perceived capacity to change, with importance rated, on average, 8 out of 10 and confidence and know-how rated 6 out of 10. This suggests that our proactive recruitment strategy can reach those who may benefit from, and be interested in, support. The relatively low baseline ratings of know-how and confidence also suggest potential to deliver effective behavior change support, as previous research shows that enhancing individuals’ confidence in their ability to change and improving their knowledge of how to change can lead to positive behavior modifications, such as reduced alcohol consumption and smoking cessation [105,106]. It should be noted that these 3 measures did not constitute a primary objective of this trial, and to reduce participant burden, we used single-item measures, which cannot capture a more nuanced understanding of confidence, importance, and know-how. A related limitation is that, because participants were not necessarily actively seeking help, baseline screening leading to inclusion may have prompted reflection on behavior for some participants, which in itself may have induced change. Notably, this occurred before randomization; thus, the consequence may be that estimates are biased toward the null.

Attrition rates were high in this study; however, they were consistent with what can be expected in remote trials with low barriers to participation [107]. Attrition analyses did not indicate marked differences across factorial conditions, thereby strengthening the internal validity of the findings. However, there was some evidence that late responders and nonresponders exhibited healthier behaviors compared with early responders. This underscores the importance of the imputed analyses in supporting the main findings.

The factorial design ensured blinding by providing every participant with some form of intervention. Although social desirability is always a concern in studies in which the objectives are clear to participants—and possibly more so when outcomes are self-reported—blinding reduces the risk of compensatory rivalry and other research participation effects [108,109]. Therefore, the risk of bias stemming from the use of self-reported measures in this study is somewhat tempered. Regarding blinding, there was a risk that participants could have deliberately or inadvertently learned about others’ participation and shared intervention content, thereby becoming partially aware of other conditions. Although we cannot determine the extent to which this occurred, we judge the risk of bias to be low. If such contamination did occur, our estimates of effectiveness are likely biased toward the null.

Finally, the factorial design used in this trial allowed us to study the individual components of Buddy; however, it limited our ability to estimate the total effect of the intervention as a whole. Therefore, future studies are needed to investigate the overall effects of the Buddy intervention. Indeed, post hoc analyses suggested that access to a greater number of components increased effectiveness with respect to fruit and vegetable consumption and physical activity.

### Conclusions

Using a randomized factorial design, we dismantled a complex digital multiple behavior intervention and provided novel insights into the effectiveness of its different components. Both marginal and synergistic effects were estimated across multiple behaviors. Our results highlight which components of digital interventions contribute to effectiveness and demonstrate that component effectiveness is heterogeneous across the targeted behaviors. The findings provide an evidence base for designing more effective digital interventions. These findings should be considered in light of the risk of bias introduced by attrition to follow-up, which was high in this effectiveness trial with low barriers to participation. The trial also relied on self-reported measures, although the risk of bias associated with this design choice may have been partially mitigated by participant blinding.

## Appendix

Multimedia Appendix 1 CONSORT-eHEALTH checklist (V 1.6.1).

Multimedia Appendix 2 Informed consent form.

Multimedia Appendix 3 Questionnaires.

Multimedia Appendix 4 Intervention description and allocation.

Multimedia Appendix 5 Attrition nonresponse.

Multimedia Appendix 6 Attrition attempts.

Multimedia Appendix 7 Complete-case estimates of effect.

Multimedia Appendix 8 Effect estimates based on imputed data.

Multimedia Appendix 9 Estimates of the effects of component count.

## Abbreviations

BCT: behavior change technique
CoI: compatibility interval
CONSORT: Consolidated Standards of Reporting Trials
IRR: incidence rate ratio
ISRCTN: International Standard Randomised Controlled Trial Number
MVPA: moderate-to-vigorous physical activity
POE: probability of effect

## Data Availability

The datasets used or analyzed in this study are available from the corresponding author (MB) upon reasonable request. Ethical approval is required for data sharing.
