# Supplementary material for: Effectiveness of the Components of a Digital Multiple Health Behavior Intervention Among University Students (Buddy): Factorial Randomized Trial
Source: J Med Internet Res. 2026 Mar 9;28:e88884. doi: 10.2196/88884 (PMC13010081; doi:10.2196/88884)
Supplement: Multimedia Appendix 5 [file jmir_v28i1e88884_app5.pdf]

APPENDIX D – ATTRITION NON-RESPONSE ANALYSES

SUMMARY

At the 2-month follow-up interval we found no strong evidence that any of the measured baseline characteristics were associated with non-response. Estimates of associations between baseline characteristics and response at 2 months without interactions between components can be found in Table 1 and with interactions in Table 2.

At the 4-month follow-up interval, we found evidence of an association between age and non-response, with older participants being more likely to respond (OR = 0.98, 95% CI = 0.96, 1.00, probability of association = 99.2%). There was also an association between the number of cigarettes smoked at baseline and non-response, with those smoking more less likely to respond (OR = 1.0095, 95% CI = 1.003; 1.017, probability of association = 99.8%). These two associations are depicted in Figure 1 (a) and (b), where it can be seen that the association regarding smoking is driven by a few data points. Interactions between components and age and cigarettes smoked at baseline showed no differential associations (Table 4).

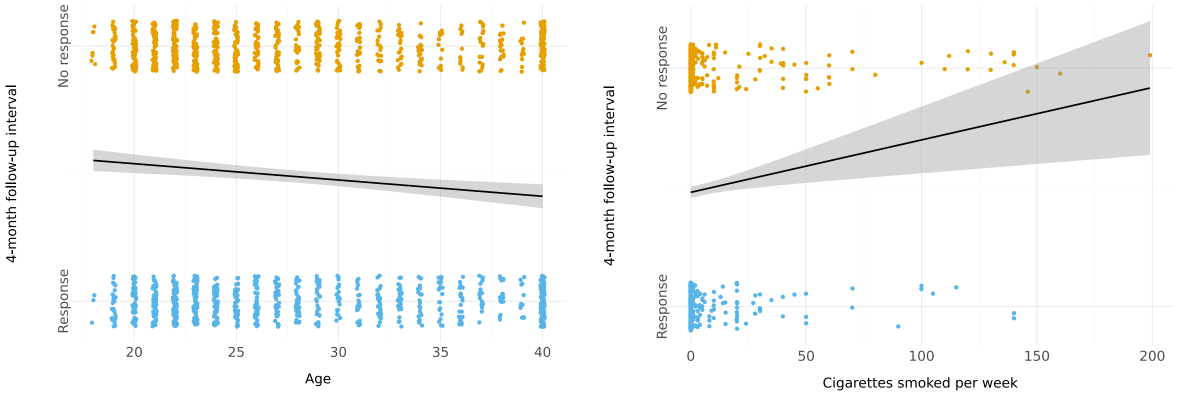

Figure 1 - Association between response to the 4-month follow-up and (a) age and (b) cigarettes smoked per week at baseline

TABLES

Table 1 – Associations between baseline characteristics and non-response to the 2-month follow-up

	Est.	Prob.
C1	1.00 (0.97; 1.03)	50.3%
C2	1.00 (0.89; 1.02)	56.6%
C3	1.00 (0.98; 1.08)	55.3%
C4	1.00 (0.99; 1.24)	59.9%
C5	1.00 (0.96; 1.02)	52.4%
C6	1.00 (0.98; 1.03)	52.1%
Man vs. Woman	1.00 (0.89; 1.02)	55.5%
Age	1.00 (0.96; 1.00)	67.6%
Heavy episodic drinking	1.01 (1.00; 1.07)	77.4%
Weekly alcohol consumption	1.00 (0.99; 1.02)	67.7%
Weekly moderate and vigorous physical activity	1.00 (1.00; 1.00)	90.6%

Average daily portions of fruit and vegetables	1.00 (0.95; 1.02)	55.4%
Average daily portions of candy and snacks	1.00 (0.99; 1.01)	65.4%
Weekly consumption of sugary drinks	1.00 (0.98; 1.01)	65.5%
Number of cigarettes smoked per week	1.00 (1.00; 1.01)	80.7%
Body mass index	1.00 (0.99; 1.01)	56.6%
Perceived stress	1.00 (0.99; 1.04)	68.4%
Importance of change	1.00 (0.99; 1.06)	64.1%
Confidence in ability to change	1.00 (0.99; 1.06)	68.6%
Knowledge of how to change	1.00 (0.99; 1.02)	54.1%

Est. – Median of the posterior distribution of odds ratios with 95% compatibility intervals.
Prob. – Proportion of the posterior distribution above or below the null in the direction of the median.

Table 2 - Associations between baseline characteristics and non-response to the 2-month follow-up with interactions between characteristics and intervention components

	**Est.**	**Prob.**
C1	1.00 (1.00; 1.00)	50.2%
C2	1.00 (1.00; 1.00)	50.8%
C3	1.00 (1.00; 1.00)	50.1%
C4	1.00 (1.00; 1.00)	50.3%
C5	1.00 (1.00; 1.00)	50.2%
C6	1.00 (1.00; 1.00)	50.0%
Man vs. Woman	1.00 (1.00; 1.00)	50.7%
Age	1.00 (1.00; 1.00)	53.7%
Heavy episodic drinking	1.00 (1.00; 1.05)	55.8%
Weekly alcohol consumption	1.00 (1.00; 1.01)	54.2%
Weekly moderate and vigorous physical activity	1.00 (1.00; 1.00)	64.8%
Average daily portions of fruit and vegetables	1.00 (1.00; 1.00)	51.2%
Average daily portions of candy and snacks	1.00 (1.00; 1.00)	53.2%
Weekly consumption of sugary drinks	1.00 (1.00; 1.00)	52.0%
Number of cigarettes smoked per week	1.00 (1.00; 1.00)	58.5%
Body mass index	1.00 (1.00; 1.00)	51.3%
Perceived stress	1.00 (1.00; 1.00)	52.0%
Importance of change	1.00 (1.00; 1.01)	51.7%
Confidence in ability to change	1.00 (1.00; 1.01)	52.8%
Knowledge of how to change	1.00 (1.00; 1.00)	50.3%
**C1 interactions**		
C1 x Man vs. Woman	1.00 (1.00; 1.00)	50.6%
C1 x Age	1.00 (1.00; 1.00)	50.4%
C1 x Heavy episodic drinking	1.00 (1.00; 1.01)	51.8%
C1 x Weekly alcohol consumption	1.00 (1.00; 1.00)	51.6%
C1 x Weekly moderate and vigorous physical activity	1.00 (1.00; 1.01)	60.6%
C1 x Average daily portions of fruit and vegetables	1.00 (1.00; 1.00)	50.4%
C1 x Average daily portions of candy and snacks	1.00 (1.00; 1.00)	50.6%
C1 x Weekly consumption of sugary drinks	1.00 (1.00; 1.00)	50.9%
C1 x Number of cigarettes smoked per week	1.00 (1.00; 1.00)	53.7%
C1 x Body mass index	1.00 (1.00; 1.00)	50.8%
C1 x Perceived stress	1.00 (1.00; 1.00)	50.3%
C1 x Importance of change	1.00 (1.00; 1.00)	50.4%
C1 x Confidence in ability to change	1.00 (1.00; 1.00)	50.8%

C1 x Knowledge of how to change	1.00 (1.00; 1.00)	50.5%
**C2 interactions**		
C2 x Man vs. Woman	1.00 (0.99; 1.00)	51.2%
C2 x Age	1.00 (1.00; 1.00)	55.6%
C2 x Heavy episodic drinking	1.00 (1.00; 1.00)	51.0%
C2 x Weekly alcohol consumption	1.00 (1.00; 1.00)	52.2%
C2 x Weekly moderate and vigorous physical activity	1.00 (1.00; 1.00)	77.0%
C2 x Average daily portions of fruit and vegetables	1.00 (1.00; 1.00)	50.2%
C2 x Average daily portions of candy and snacks	1.00 (1.00; 1.00)	52.1%
C2 x Weekly consumption of sugary drinks	1.00 (1.00; 1.00)	52.0%
C2 x Number of cigarettes smoked per week	1.00 (1.00; 1.00)	53.4%
C2 x Body mass index	1.00 (1.00; 1.00)	54.6%
C2 x Perceived stress	1.00 (1.00; 1.00)	50.3%
C2 x Importance of change	1.00 (1.00; 1.00)	51.3%
C2 x Confidence in ability to change	1.00 (1.00; 1.00)	50.4%
C2 x Knowledge of how to change	1.00 (1.00; 1.00)	51.6%
**C3 interactions**		
C3 x Man vs. Woman	1.00 (1.00; 1.00)	50.2%
C3 x Age	1.00 (1.00; 1.00)	52.6%
C3 x Heavy episodic drinking	1.00 (1.00; 1.06)	55.6%
C3 x Weekly alcohol consumption	1.00 (1.00; 1.01)	53.0%
C3 x Weekly moderate and vigorous physical activity	1.00 (1.00; 1.00)	73.1%
C3 x Average daily portions of fruit and vegetables	1.00 (1.00; 1.00)	50.5%
C3 x Average daily portions of candy and snacks	1.00 (1.00; 1.00)	51.9%
C3 x Weekly consumption of sugary drinks	1.00 (1.00; 1.00)	50.7%
C3 x Number of cigarettes smoked per week	1.00 (1.00; 1.00)	50.8%
C3 x Body mass index	1.00 (1.00; 1.00)	52.8%
C3 x Perceived stress	1.00 (1.00; 1.00)	52.0%
C3 x Importance of change	1.00 (1.00; 1.00)	52.0%
C3 x Confidence in ability to change	1.00 (1.00; 1.01)	53.0%
C3 x Knowledge of how to change	1.00 (1.00; 1.00)	51.3%
**C4 interactions**		
C4 x Man vs. Woman	1.00 (1.00; 1.00)	50.5%
C4 x Age	1.00 (1.00; 1.01)	57.8%
C4 x Heavy episodic drinking	1.00 (1.00; 1.00)	52.1%
C4 x Weekly alcohol consumption	1.00 (1.00; 1.01)	52.9%
C4 x Weekly moderate and vigorous physical activity	1.00 (1.00; 1.00)	53.0%
C4 x Average daily portions of fruit and vegetables	1.00 (1.00; 1.00)	50.2%
C4 x Average daily portions of candy and snacks	1.00 (1.00; 1.00)	51.9%
C4 x Weekly consumption of sugary drinks	1.00 (1.00; 1.00)	50.7%
C4 x Number of cigarettes smoked per week	1.00 (1.00; 1.00)	52.4%
C4 x Body mass index	1.00 (1.00; 1.01)	60.0%
C4 x Perceived stress	1.00 (1.00; 1.04)	57.7%
C4 x Importance of change	1.00 (1.00; 1.01)	54.3%
C4 x Confidence in ability to change	1.00 (1.00; 1.01)	53.0%
C4 x Knowledge of how to change	1.00 (1.00; 1.00)	51.4%
**C5 interactions**		
C5 x Man vs. Woman	1.00 (1.00; 1.00)	50.1%
C5 x Age	1.00 (1.00; 1.00)	54.5%
C5 x Heavy episodic drinking	1.00 (1.00; 1.01)	52.8%
C5 x Weekly alcohol consumption	1.00 (1.00; 1.01)	53.8%

C5 x Weekly moderate and vigorous physical activity	1.00 (1.00; 1.00)	76.3%
C5 x Average daily portions of fruit and vegetables	1.00 (1.00; 1.00)	50.4%
C5 x Average daily portions of candy and snacks	1.00 (1.00; 1.00)	51.4%
C5 x Weekly consumption of sugary drinks	1.00 (1.00; 1.00)	52.4%
C5 x Number of cigarettes smoked per week	1.00 (1.00; 1.00)	51.6%
C5 x Body mass index	1.00 (1.00; 1.00)	55.2%
C5 x Perceived stress	1.00 (1.00; 1.00)	50.4%
C5 x Importance of change	1.00 (1.00; 1.00)	51.0%
C5 x Confidence in ability to change	1.00 (1.00; 1.00)	50.7%
C5 x Knowledge of how to change	1.00 (1.00; 1.00)	51.2%
**C6 interactions**		
C6 x Man vs. Woman	1.00 (1.00; 1.00)	50.1%
C6 x Age	1.00 (1.00; 1.00)	50.7%
C6 x Heavy episodic drinking	1.00 (1.00; 1.01)	53.1%
C6 x Weekly alcohol consumption	1.00 (1.00; 1.00)	51.1%
C6 x Weekly moderate and vigorous physical activity	1.00 (1.00; 1.00)	74.0%
C6 x Average daily portions of fruit and vegetables	1.00 (1.00; 1.00)	50.4%
C6 x Average daily portions of candy and snacks	1.00 (1.00; 1.00)	51.3%
C6 x Weekly consumption of sugary drinks	1.00 (1.00; 1.00)	52.1%
C6 x Number of cigarettes smoked per week	1.00 (1.00; 1.00)	52.3%
C6 x Body mass index	1.00 (1.00; 1.00)	51.5%
C6 x Perceived stress	1.00 (1.00; 1.00)	50.5%
C6 x Importance of change	1.00 (1.00; 1.00)	50.2%
C6 x Confidence in ability to change	1.00 (1.00; 1.00)	51.2%
C6 x Knowledge of how to change	1.00 (1.00; 1.00)	50.4%

Est. – Median of the posterior distribution of odds ratios with 95% compatibility intervals.
Prob. – Proportion of the posterior distribution above or below the null in the direction of the median.

Table 3 - Associations between baseline characteristics and non-response to the 4-month follow-up

	Est.	Prob.
C1	1.00 (0.96; 1.09)	55.5%
C2	1.00 (0.97; 1.17)	62.0%
C3	1.00 (0.97; 1.13)	59.9%
C4	1.02 (0.99; 1.44)	78.3%
C5	1.00 (0.89; 1.04)	57.9%
C6	1.00 (0.96; 1.08)	54.6%
Man vs. Woman	0.99 (0.66; 1.02)	71.1%
Age	0.98 (0.97; 1.00)	99.2%
Heavy episodic drinking	1.03 (1.00; 1.09)	90.1%
Weekly alcohol consumption	1.01 (0.99; 1.04)	83.1%
Weekly moderate and vigorous physical activity	1.00 (1.00; 1.00)	91.2%
Average daily portions of fruit and vegetables	1.00 (0.96; 1.04)	51.3%
Average daily portions of candy and snacks	1.00 (1.00; 1.01)	58.2%
Weekly consumption of sugary drinks	1.00 (0.99; 1.02)	66.7%
Number of cigarettes smoked per week	1.01 (1.00; 1.02)	99.8%
Body mass index	1.00 (0.99; 1.02)	54.9%
Perceived stress	1.00 (0.99; 1.03)	66.1%
Importance of change	1.00 (0.98; 1.05)	67.0%
Confidence in ability to change	1.01 (0.99; 1.06)	76.1%

Knowledge of how to change	0.99 (0.97; 1.02)	60.0%
**Est.** – Median of the posterior distribution of odds ratios with 95% compatibility intervals.<br>**Prob.** – Proportion of the posterior distribution above or below the null in the direction of the median.		

Table 4 - Associations between baseline characteristics and non-response to the 4-month follow-up with interactions between characteristics and intervention components

	Est.	Prob.
C1	1.00 (1.00; 1.00)	50.2%
C2	1.00 (1.00; 1.01)	50.6%
C3	1.00 (1.00; 1.01)	50.5%
C4	1.00 (1.00; 1.01)	51.6%
C5	1.00 (1.00; 1.00)	50.1%
C6	1.00 (1.00; 1.00)	50.4%
Man vs. Woman	1.00 (1.00; 1.00)	51.9%
Age	0.99 (0.97; 1.00)	82.9%
Heavy episodic drinking	1.00 (1.00; 1.09)	66.1%
Weekly alcohol consumption	1.00 (1.00; 1.03)	61.1%
Weekly moderate and vigorous physical activity	1.00 (1.00; 1.00)	62.9%
Average daily portions of fruit and vegetables	1.00 (1.00; 1.00)	50.1%
Average daily portions of candy and snacks	1.00 (1.00; 1.00)	51.5%
Weekly consumption of sugary drinks	1.00 (1.00; 1.01)	53.6%
Number of cigarettes smoked per week	1.00 (1.00; 1.02)	86.3%
Body mass index	1.00 (1.00; 1.00)	51.1%
Perceived stress	1.00 (1.00; 1.01)	52.0%
Importance of change	1.00 (1.00; 1.01)	51.9%
Confidence in ability to change	1.00 (1.00; 1.01)	53.2%
Knowledge of how to change	1.00 (1.00; 1.00)	51.7%
**C1 interactions**		
C1 x Man vs. Woman	1.00 (0.99; 1.00)	50.5%
C1 x Age	1.00 (1.00; 1.00)	50.7%
C1 x Heavy episodic drinking	1.00 (1.00; 1.01)	51.7%
C1 x Weekly alcohol consumption	1.00 (1.00; 1.00)	50.7%
C1 x Weekly moderate and vigorous physical activity	1.00 (1.00; 1.00)	83.7%
C1 x Average daily portions of fruit and vegetables	1.00 (1.00; 1.00)	50.0%
C1 x Average daily portions of candy and snacks	1.00 (1.00; 1.00)	50.6%
C1 x Weekly consumption of sugary drinks	1.00 (1.00; 1.01)	52.8%
C1 x Number of cigarettes smoked per week	1.00 (1.00; 1.01)	55.3%
C1 x Body mass index	1.00 (1.00; 1.00)	50.2%
C1 x Perceived stress	1.00 (1.00; 1.00)	51.2%
C1 x Importance of change	1.00 (1.00; 1.00)	51.0%
C1 x Confidence in ability to change	1.00 (1.00; 1.01)	52.7%
C1 x Knowledge of how to change	1.00 (1.00; 1.00)	50.1%
**C2 interactions**		
C2 x Man vs. Woman	1.00 (0.99; 1.00)	51.3%
C2 x Age	1.00 (1.00; 1.01)	56.5%
C2 x Heavy episodic drinking	1.00 (1.00; 1.01)	51.6%
C2 x Weekly alcohol consumption	1.00 (1.00; 1.02)	56.4%
C2 x Weekly moderate and vigorous physical activity	1.00 (1.00; 1.00)	51.1%

C2 x Average daily portions of fruit and vegetables	1.00 (1.00; 1.00)	50.2%
C2 x Average daily portions of candy and snacks	1.00 (1.00; 1.00)	51.1%
C2 x Weekly consumption of sugary drinks	1.00 (1.00; 1.00)	50.3%
C2 x Number of cigarettes smoked per week	1.00 (1.00; 1.01)	58.5%
C2 x Body mass index	1.00 (1.00; 1.01)	60.6%
C2 x Perceived stress	1.00 (1.00; 1.01)	55.8%
C2 x Importance of change	1.00 (1.00; 1.02)	57.2%
C2 x Confidence in ability to change	1.00 (1.00; 1.02)	57.0%
C2 x Knowledge of how to change	1.00 (1.00; 1.01)	51.2%
**C3 interactions**		
C3 x Man vs. Woman	1.00 (0.99; 1.00)	50.4%
C3 x Age	1.00 (1.00; 1.00)	53.3%
C3 x Heavy episodic drinking	1.00 (1.00; 1.03)	54.4%
C3 x Weekly alcohol consumption	1.00 (1.00; 1.02)	56.0%
C3 x Weekly moderate and vigorous physical activity	1.00 (1.00; 1.00)	63.9%
C3 x Average daily portions of fruit and vegetables	1.00 (1.00; 1.01)	50.1%
C3 x Average daily portions of candy and snacks	1.00 (1.00; 1.00)	50.1%
C3 x Weekly consumption of sugary drinks	1.00 (1.00; 1.01)	53.2%
C3 x Number of cigarettes smoked per week	1.00 (1.00; 1.00)	50.6%
C3 x Body mass index	1.00 (1.00; 1.01)	57.2%
C3 x Perceived stress	1.00 (1.00; 1.01)	53.3%
C3 x Importance of change	1.00 (1.00; 1.01)	54.2%
C3 x Confidence in ability to change	1.00 (1.00; 1.02)	56.1%
C3 x Knowledge of how to change	1.00 (1.00; 1.01)	51.4%
**C4 interactions**		
C4 x Man vs. Woman	1.00 (1.00; 1.00)	50.2%
C4 x Age	1.00 (1.00; 1.01)	59.8%
C4 x Heavy episodic drinking	1.00 (1.00; 1.01)	53.0%
C4 x Weekly alcohol consumption	1.00 (1.00; 1.01)	54.2%
C4 x Weekly moderate and vigorous physical activity	1.00 (1.00; 1.00)	70.8%
C4 x Average daily portions of fruit and vegetables	1.00 (1.00 1.01)	50.3%
C4 x Average daily portions of candy and snacks	1.00 (1.00; 1.01)	53.6%
C4 x Weekly consumption of sugary drinks	1.00 (1.00; 1.01)	53.0%
C4 x Number of cigarettes smoked per week	1.00 (1.00; 1.01)	57.4%
C4 x Body mass index	1.00 (1.00; 1.02)	71.5%
C4 x Perceived stress	1.00 (1.00; 1.02)	57.4%
C4 x Importance of change	1.00 (1.00; 1.02)	55.7%
C4 x Confidence in ability to change	1.00 (1.00; 1.02)	55.3%
C4 x Knowledge of how to change	1.00 (1.00; 1.01)	53.1%
**C5 interactions**		
C5 x Man vs. Woman	1.00 (1.00; 1.00)	50.7%
C5 x Age	1.00 (0.99; 1.00)	65.1%
C5 x Heavy episodic drinking	1.00 (1.00; 1.03)	54.0%
C5 x Weekly alcohol consumption	1.00 (1.00; 1.03)	57.8%
C5 x Weekly moderate and vigorous physical activity	1.00 (1.00; 1.00)	72.7%
C5 x Average daily portions of fruit and vegetables	1.00 (1.00; 1.00)	50.8%
C5 x Average daily portions of candy and snacks	1.00 (1.00; 1.00)	50.6%
C5 x Weekly consumption of sugary drinks	1.00 (1.00; 1.00)	50.2%
C5 x Number of cigarettes smoked per week	1.00 (1.00; 1.01)	54.9%
C5 x Body mass index	1.00 (0.99; 1.00)	59.3%
C5 x Perceived stress	1.00 (1.00; 1.00)	50.1%

C5 x Importance of change	1.00 (1.00; 1.00)	51.8%
C5 x Confidence in ability to change	1.00 (1.00; 1.01)	50.5%
C5 x Knowledge of how to change	1.00 (0.99; 1.00)	53.0%
**C6 interactions**		
C6 x Man vs. Woman	1.00 (0.99; 1.00)	50.7%
C6 x Age	1.00 (1.00; 1.00)	52.9%
C6 x Heavy episodic drinking	1.00 (1.00; 1.10)	61.0%
C6 x Weekly alcohol consumption	1.00 (1.00; 1.01)	53.8%
C6 x Weekly moderate and vigorous physical activity	1.00 (1.00; 1.00)	71.1%
C6 x Average daily portions of fruit and vegetables	1.00 (1.00; 1.01)	50.6%
C6 x Average daily portions of candy and snacks	1.00 (0.99; 1.00)	54.1%
C6 x Weekly consumption of sugary drinks	1.00 (1.00; 1.00)	50.1%
C6 x Number of cigarettes smoked per week	1.00 (1.00; 1.00)	52.2%
C6 x Body mass index	1.00 (1.00; 1.00)	51.3%
C6 x Perceived stress	1.00 (1.00; 1.00)	51.8%
C6 x Importance of change	1.00 (1.00; 1.00)	50.4%
C6 x Confidence in ability to change	1.00 (1.00; 1.01)	51.8%
C6 x Knowledge of how to change	1.00 (1.00; 1.00)	50.7%

Est. – Median of the posterior distribution of odds ratios with 95% compatibility intervals.

Prob. – Proportion of the posterior distribution above or below the null in the direction of the median.
