# Supplementary material for: Effectiveness of the Components of a Digital Multiple Health Behavior Intervention Among University Students (Buddy): Factorial Randomized Trial
Source: J Med Internet Res. 2026 Mar 9;28:e88884. doi: 10.2196/88884 (PMC13010081; doi:10.2196/88884)
Supplement: Multimedia Appendix 6 [file jmir_v28i1e88884_app6.pdf]

APPENDIX E – ATTRITION ATTEMPTS ANALYSES

SUMMARY

The most consistent evidence for any differentiation between early and late responders was found with respect to alcohol consumption and fruit and vegetables. At the 4-month follow-up interval, weekly alcohol consumption was generally lower among later responders, however, not so for participants with access to C1. At the same time there was also evidence that heavy episodic drinking was more frequent among late responders with access to C1 at the 4-month interval. Fruit and vegetable consumption was higher among late responders at the 2-month interval, with a slight attenuation among those with access to C1. At the 4-month interval there was somewhat weaker evidence of higher fruit and vegetable consumption among late responders generally. There was additional evidence that sugary drinks consumption was higher among late responders with access to C1 specifically, and that smoking cessation was more common among late responders with access to C1.

Overall, the evidence indicates that late responders had somewhat more healthy behaviors with respect to alcohol and fruit and vegetables, and that these associations were attenuated for participants with access to C1. While the degree to which this is due to chance is not possible to say, under the assumption that late responders are more alike non-responders than early responders, the evidence may indicate systematic attrition.

TABLES

WEEKLY ALCOHOL CONSUMPTION

Table 1 - Associations between weekly alcohol consumption and attempts to collect follow-up at the 2- and 4-month follow-up interval

	**Est.**	**Prob.**
**2-month follow-up**		
Attempts	0.95 (0.70; 1.32)	62.2%
C1 x Attempts	1.01 (0.80; 1.27)	53.8%
C2 x Attempts	0.99 (0.78; 1.26)	53.7%
C3 x Attempts	0.90 (0.71; 1.13)	83.4%
C4 x Attempts	1.17 (0.93; 1.47)	91.8%
C5 x Attempts	1.03 (0.83; 1.28)	59.9%
C6 x Attempts	0.96 (0.75; 1.23)	62.6%
**4-month follow-up**		
Attempts	0.76 (0.56; 1.04)	95.8%
C1 x Attempts	1.37 (1.04; 1.78)	98.8%
C2 x Attempts	1.01 (0.80; 1.27)	54.6%
C3 x Attempts	1.17 (0.91; 1.50)	88.6%
C4 x Attempts	1.01 (0.77; 1.31)	52.4%
C5 x Attempts	0.85 (0.68; 1.08)	91.1%
C6 x Attempts	1.13 (0.89; 1.43)	84.0%

Est. – Median of the posterior distribution of incidence rate ratios with 95% compatibility intervals.
Prob. – Proportion of the posterior distribution above or below the null in the direction of the median.

HEAVY EPISODIC DRINKING

Table 2 - Associations between monthly episodes of heavy drinking and attempts to collect follow-up at the 2- and 4-month follow-up interval

	Est.	Prob.
**2-month follow-up**		
Attempts	1.02 (0.93; 1.12)	67.8%
C1 x Attempts	1.03 (0.95; 1.11)	74.6%
C2 x Attempts	1.03 (0.95; 1.11)	74.7%
C3 x Attempts	1.00 (0.93; 1.07)	53.4%
C4 x Attempts	1.02 (0.94; 1.10)	65.2%
C5 x Attempts	0.94 (0.87; 1.01)	95.2%
C6 x Attempts	1.02 (0.94; 1.10)	68.9%
**4-month follow-up**		
Attempts	1.01 (0.89; 1.15)	56.5%
C1 x Attempts	1.12 (1.01; 1.25)	98.0%
C2 x Attempts	0.96 (0.86; 1.07)	78.2%
C3 x Attempts	1.00 (0.89; 1.11)	53.3%
C4 x Attempts	0.91 (0.81; 1.01)	96.5%
C5 x Attempts	0.96 (0.86; 1.06)	79.4%
C6 x Attempts	0.99 (0.88; 1.09)	65.4%
**Est.** – Median of the posterior distribution of incidence rate ratios with 95% compatibility intervals.<br>**Prob.** – Proportion of the posterior distribution above or below the null in the direction of the median.		

FRUIT AND VEGETABLES

Table 3 – Associations between average daily portions of fruit and vegetables and attempts to collect follow-up at the 2- and 4-month follow-up intervals

	Est.	Prob.
**2-month follow-up**		
Attempts	0.15 (0.06; 0.22)	> 99.9%
C1 x Attempts	-0.06 (-0.13; 0.00)	97.7%
C2 x Attempts	-0.01 (-0.07; 0.06)	60.7%
C3 x Attempts	-0.01 (-0.08; 0.05)	65.9%
C4 x Attempts	-0.03 (-0.10; 0.03)	84.4%
C5 x Attempts	-0.00 (-0.07; 0.06)	53.6%
C6 x Attempts	-0.02 (-0.08; 0.04)	73.9%
**4-month follow-up**		
Attempts	0.08 (-0.02; 0.18)	95.1%
C1 x Attempts	-0.03 (-0.11; 0.04)	79.7%
C2 x Attempts	0.02 (-0.06; 0.10)	69.8%
C3 x Attempts	-0.05 (-0.13; 0.02)	91.4%
C4 x Attempts	-0.01 (-0.08; 0.07)	58.1%
C5 x Attempts	0.01 (-0.07; 0.09)	63.2%
C6 x Attempts	-0.01 (-0.09; 0.07)	61.1%
**Est.** – Median of the posterior distribution of linear associations with 95% compatibility intervals.<br>**Prob.** – Proportion of the posterior distribution above or below the null in the direction of the median.		

SUGARY DRINKS

Table 4 - Associations between weekly consumption of sugary drinks and attempts to collect follow-up at the 2- and 4-month follow-up interval

	Est.	Prob.
**2-month follow-up**		
Attempts	1.09 (0.99; 1.21)	95.6%
C1 x Attempts	1.02 (0.94; 1.11)	67.1%
C2 x Attempts	1.03 (0.95; 1.12)	76.4%
C3 x Attempts	1.02 (0.97; 1.10)	64.3%
C4 x Attempts	0.98 (0.90; 1.07)	68.8%
C5 x Attempts	0.97 (0.89; 1.05)	77.8%
C6 x Attempts	0.98 (0.90; 1.06)	72.9%
**4-month follow-up**		
Attempts	0.99 (0.88; 1.11)	58.1%
C1 x Attempts	1.16 (1.04; 1.26)	99.8%
C2 x Attempts	1.05 (0.95; 1.16)	83.9%
C3 x Attempts	1.06 (0.96; 1.17)	86.6%
C4 x Attempts	0.97 (0.88; 1.07)	72.2%
C5 x Attempts	0.94 (0.86; 1.04)	89.6%
C6 x Attempts	1.00 (0.91; 1.11)	52.2%

Est. – Median of the posterior distribution of incidence rate ratios with 95% compatibility intervals.
Prob. – Proportion of the posterior distribution above or below the null in the direction of the median.

MODERATE AND VIGOROUS PHYSICAL ACTIVITY

Table 5 - Associations between weekly moderate and physical activity and attempts to collect follow-up at the 2- and 4-month follow-up interval

	Est.	Prob.
**2-month follow-up**		
Attempts	19.9 (-4.44; 43.8)	94.6%
C1 x Attempts	-4.1 (-22.6; 14.7)	66.6%
C2 x Attempts	-5.5 (-23.9; 13.0)	72.2%
C3 x Attempts	11.1 (-7.9; 29.8)	87.4%
C4 x Attempts	-10.4 (-28.9; 8.4)	86.2%
C5 x Attempts	4.0 (-14.3; 23.1)	66.1%
C6 x Attempts	-8.8 (-27.4; 9.9)	82.0%
**4-month follow-up**		
Attempts	16.6 (-13.3; 46.5)	86.0%
C1 x Attempts	-3.5 (-26.5; 19.2)	62.0%
C2 x Attempts	12.0 (-11.9; 36.2)	83.7%
C3 x Attempts	-11.8 (-34.9; 10.8)	84.5%
C4 x Attempts	4.8 (-17.9; 27.7)	66.1%
C5 x Attempts	13.3 (-9.2; 35.9)	87.4%
C6 x Attempts	-8.5 (-31.3; 14.1)	77.2%

Est. – Median of the posterior distribution of linear associations with 95% compatibility intervals.
Prob. – Proportion of the posterior distribution above or below the null in the direction of the median.

SMOKING CESSATION

Table 6 - Associations between smoking cessation and attempts to collect follow-up at the 2- and 4-month follow-up intervals

	**Est.**	**Prob.**
**2-month follow-up**		
Attempts	0.95 (0.64; 1.42)	59.8%
C1 x Attempts	1.04 (0.72; 1.50)	59.0%
C2 x Attempts	0.93 (0.65; 1.32)	66.4%
C3 x Attempts	0.97 (0.66; 1.40)	57.1%
C4 x Attempts	0.94 (0.64; 1.36)	63.8%
C5 x Attempts	0.75 (0.52; 1.08)	94.4%
C6 x Attempts	1.41 (0.96; 2.10)	96.3%
**4-month follow-up**		
Attempts	0.82 (0.54; 1.23)	82.6%
C1 x Attempts	1.45 (1.00; 2.15)	97.5%
C2 x Attempts	1.17 (0.80; 1.71)	78.7%
C3 x Attempts	1.31 (0.90; 1.93)	92.3%
C4 x Attempts	1.05 (0.70; 1.56)	59.6%
C5 x Attempts	0.90 (0.60; 1.33)	71.3%
C6 x Attempts	0.85 (0.58; 1.25)	79.0%

Est. – Median of the posterior distribution of odds ratios with 95% compatibility intervals.
Prob. – Proportion of the posterior distribution above or below the null in the direction of the median.
