# Supplementary material for: Effectiveness of the Components of a Digital Multiple Health Behavior Intervention Among University Students (Buddy): Factorial Randomized Trial
Source: J Med Internet Res. 2026 Mar 9;28:e88884. doi: 10.2196/88884 (PMC13010081; doi:10.2196/88884)
Supplement: Multimedia Appendix 7 [file jmir_v28i1e88884_app7.pdf]

APPENDIX F – ESTIMATES OF EFFECTS (COMPLETE CASES)

The tables in this appendix present the estimated effects of individual and pairwise combinations of components on primary and secondary outcome measures. The analyses were done using complete case data. The first part of each table shows estimates of effects for the individual components at each follow-up interval. The second part of the table, under the heading “two-way interactions”, shows the estimated effects of pairwise combinations of components.

For primary outcomes, analyses were among those who were indicated as having unhealthy behaviors at baseline, for example, those included in analyses of outcomes of moderate and vigorous physical activity were those who had less then 150 minutes at baseline.

Numbers included in the complete-case analyses:

Total weekly alcohol consumption: 107 (2-month); 80 (4-month)
Heavy episodic drinking: 624 (2-month); 461 (4-month)
Daily portions of fruit and vegetables: 1108 (2-month); 862 (4-month)
Weekly consumption of sugary drinks: 534 (2-month); 397 (4-month)
Weekly moderate to vigorous physical activity: 564 (2-month); 450 (4-month)
Four week point prevalence of smoking cessation: 128 (2-month); 97 (4-month)
Cigarettes smoked weekly (among current smokers): 84 (2-month); 63 (4-month)
Candy and snacks: 1114 (2-month); 873 (4-month)
Stress: 1084 (2-month); 861 (4-month)
Body mass index: 1100 (2-month); 870 (4-month)

Table 1 - Estimates of effects of components, including two-way interactions among components, on total weekly alcohol consumption at 2- and 4-month follow-up.

	C1		C2		C3		C4		C5		C6	
	**Est.**	**Prob.**	**Est.**	**Prob.**	**Est.**	**Prob.**	**Est.**	**Prob.**	**Est.**	**Prob.**	**Est.**	**Prob.**
**2-month**	1.06 (0.77; 1.50)	63.7%	0.95 (0.68; 1.32)	63.1%	0.79 (0.58; 1.08)	93.6%	0.91 (0.66; 1.25)	71.9%	0.98 (0.72; 1.34)	53.9%	0.90 (0.65; 1.23)	75.5%
**4-month**	1.01 (0.71; 1.45)	53.1%	1.06 (0.74; 1.53)	63.4%	0.87 (0.62; 1.22)	79.6%	0.96 (0.67; 1.36)	59.9%	1.19 (0.84; 1.69)	83.4%	0.65 (0.45; 0.93)	99.1%
**Two-way interactions**												
**C2**												
**2-month**	1.01 (0.68; 1.51)	51.7%										
**4-month**	1.10 (0.70; 1.75)	66.1%										
**C3**												
**2-month**	0.83 (0.52; 1.33)	78.0%	0.75 (0.49; 1.16)	90.2%								
**4-month**	0.87 (0.54; 1.39)	72.1%	0.92 (0.56; 1.55)	61.9%								
**C4**												
**2-month**	0.97 (0.63; 1.49)	56.4%	0.87 (0.51; 1.45)	70.8%	0.73 (0.47; 1.14)	91.8%						
**4-month**	0.91 (0.55; 1.53)	63.6%	1.02 (0.58; 1.76)	52.1%	0.86 (0.51; 1.41)	72.6%						
**C5**												
**2-month**	1.02 (0.65; 1.61)	53.5%	0.94 (0.62; 1.43)	61.3%	0.76 (0.48; 1.23)	87.2%	0.93 (0.61; 1.42)	64.3%				
**4-month**	1.19 (0.75; 1.93)	76.9%	1.24 (0.75; 2.05)	79.5%	1.02 (0.62; 1.69)	52.8%	1.18 (0.74; 1.90)	75.7%				
**C6**												
**2-month**	0.88 (0.56; 1.37)	72.0%	0.84 (0.53; 1.34)	77.5%	0.66 (0.41; 1.05)	96.1%	0.83 (0.52; 1.34)	77.7%	0.83 (0.53; 1.31)	79.1%		
**4-month**	0.63 (0.37; 1.07)	95.5%	0.67 (0.42; 1.08)	95.1%	0.53 (0.31; 0.88)	99.4%	0.61 (0.36; 1.03)	96.7%	0.78 (0.47; 1.27)	84.6%		

Est. – Median of the posterior distribution of incidence rate ratios with 95% compatibility intervals.

Prob. – Proportion of the posterior distribution above or below the null in the direction of the median.

Table 2 - Estimates of effects of components, including two-way interactions among components, on frequency of heavy episodic drinking at 2- and 4-month follow-up.

	C1		C2		C3		C4		C5		C6	
	**Est.**	**Prob.**	**Est.**	**Prob.**	**Est.**	**Prob.**	**Est.**	**Prob.**	**Est.**	**Prob.**	**Est.**	**Prob.**
**2-month**	0.87 (0.74; 1.02)	95.2%	0.89 (0.75; 1.04)	93.1%	0.96 (0.82; 1.13)	68.7%	1.00 (0.86; 1.18)	51.7%	0.89 (0.76; 1.04)	92.5%	1.14 (0.98; 1.34)	95.0%
**4-month**	0.97 (0.80; 1.16)	65.2%	1.09 (0.91; 1.32)	82.8%	1.18 (0.99; 1.42)	96.5%	1.03 (0.86; 1.25)	64.2%	1.11 (0.92; 1.33)	86.1%	0.93 (0.77; 1.12)	78.6%
**Two-way interactions**												
**C2**												
**2-month**	0.78 (0.62; 0.98)	98.4%										
**4-month**	1.06 (0.81; 1.39)	66.8%										
**C3**												
**2-month**	0.84 (0.66; 1.05)	93.6%	0.85 (0.68; 1.06)	93.0%								
**4-month**	1.14 (0.89; 1.48)	84.5%	1.29 (1.00; 1.66)	97.6%								
**C4**												
**2-month**	0.88 (0.70; 1.09)	87.7%	0.88 (0.70; 1.12)	85.0%	0.97 (0.78; 1.21)	59.7%						
**4-month**	1.00 (0.78; 1.30)	51.0%	1.12 (0.86; 1.47)	79.9%	1.23 (0.95; 1.59)	93.9%						
**C5**												
**2-month**	0.78 (0.62; 0.98)	98.4%	0.79 (0.63; 0.98)	98.3%	0.85 (0.67; 1.08)	91.6%	0.90 (0.72; 1.13	82.8%				
**4-month**	1.06 (0.81; 1.37)	66.9%	1.21 (0.93; 1.56)	92.5%	1.28 (0.99; 1.64)	97.1%	1.14 (0.89; 1.50)	84.7%				
**C6**												
**2-month**	1.00 (0.80; 1.26)	50.8%	1.01 (0.81; 1.30)	54.5%	1.10 (0.89; 1.37)	79.6%	1.15 (0.92; 1.43)	88.7%	1.02 (0.81; 1.27)	56.1%		
**4-month**	0.90 (0.69; 1.16)	78.7%	1.02 (0.79; 1.31)	55.8%	1.10 (0.85; 1.42)	77.0%	0.96 (0.73; 1.25)	63.3%	1.03 (0.79; 1.36)	59.5%		

Est. – Median of the posterior distribution of incidence rate ratios with 95% compatibility intervals.
Prob. – Proportion of the posterior distribution above or below the null in the direction of the median.

Table 3 - Estimates of effects of components, including two-way interactions among components, on daily portions of fruit and vegetables consumption at 2- and 4-month follow-up.

	C1		C2		C3		C4		C5		C6	
	Est.	Prob.	Est.	Prob.	Est.	Prob.	Est.	Prob.	Est.	Prob.	Est.	Prob.
**2-month**	0.11 (-0.02; 0.24)	94.7%	-0.09 (-0.22; 0.04)	90.2%	0.09 (-0.04; 0.23)	92.0%	0.19 (0.06; 0.33)	99.8%	0.03 (-0.11; 0.16)	64.7%	-0.01 (-0.14; 0.12)	54.9%
**4-month**	0.12 (-0.03; 0.26)	94.4%	-0.01 (-0.15; 0.14)	53.6%	0.12 (-0.02; 0.27)	95.4%	0.14 (-0.01; 0.28)	96.9%	-0.05 (-0.19; 0.09)	74.9%	-0.09 (-0.23; 0.05)	88.2%
**Two-way interactions**												
**C2**												
**2-month**	0.02 (-0.16; 0.21)	58.3%										
**4-month**	0.10 (-0.10; 0.31)	83.8%										
**C3**												
**2-month**	0.20 (0.02; 0.38)	98.2%	0.01 (-0.18; 0.19)	52.0%								
**4-month**	0.24 (0.04 0.44)	99.0%	0.11 (-0.09; 0.31)	86.0%								
**C4**												
**2-month**	0.30 (0.11; 0.48)	99.9%	0.11 (-0.08; 0.29)	86.7%	0.29 (0.10; 0.47)	99.9%						
**4-month**	0.26 (0.05; 0.46)	99.4%	0.13 (-0.08; 0.34)	88.2%	0.26 (0.05; 0.46)	99.3%						
**C5**												
**2-month**	0.13 (-0.06; 0.32)	91.8%	-0.06 (-0.24; 0.12)	74.2%	0.12 (-0.07; 0.30)	88.7%	0.22 (0.04; 0.40)	99.1%				
**4-month**	0.07 (-0.14; 0.27)	74.5%	-0.06 (-0.26; 0.15)	70.4%	0.07 (-0.13; 0.28)	75.5%	0.09 (-0.12; 0.29)	79.7%				
**C6**												
**2-month**	0.10 (-0.09; 0.28)	84.9%	-0.10 (-0.28; 0.09)	84.5%	0.08 (-0.10; 0.27)	81.3%	0.18 (-0.00; 0.37)	97.5%	0.02 (-0.17; 0.20)	56.9%		
**4-month**	0.03 (-0.17; 0.24)	62.7%	-0.09 (-0.30; 0.11)	81.9%	0.04 (-0.17; 0.24)	63.1%	0.05 (-0.16; 0.26)	68.9%	-0.14 (-0.34; 0.07)	90.8%		

Est. – Median of the posterior distribution of mean differences with 95% compatibility intervals.

Prob. – Proportion of the posterior distribution above or below the null in the direction of the median.

Table 4 - Estimates of effects of components, including two-way interactions among components, on weekly sugary drinks consumption at 2- and 4-month follow-up.

	C1		C2		C3		C4		C5		C6	
	Est.	Prob.	Est.	Prob.	Est.	Prob.	Est.	Prob.	Est.	Prob.	Est.	Prob.
2-month	0.91 (0.77; 1.06)	88.4%	1.03 (0.87; 1.21)	61.2%	0.98 (0.84; 1.15)	59.2%	1.04 (0.88; 1.22)	66.5%	0.94 (0.80; 1.10)	78.8%	1.24 (1.05; 1.46)	99.4%
4-month	1.03 (0.86; 1.24)	62.2%	1.05 (0.87; 1.26)	68.8%	1.09 (0.90; 1.31)	81.5%	1.06 (0.88; 1.27)	74.2%	1.07 (0.89; 1.29)	76.6%	1.16 (0.96; 1.39)	93.8%
**Two-way interactions**												
C2												
2-month	0.93 (0.74; 1.17)	73.0%										
4-month	1.08 (0.84; 1.39)	72.4%										
C3												
2-month	0.89 (0.71; 1.11)	84.9%	1.01 (0.80; 1.27)	51.9%								
4-month	1.12 (0.87; 1.45)	80.3%	1.14 (0.89; 1.47)	84.4%								
C4												
2-month	0.95 (0.75; 1.19)	67.2%	1.06 (0.83; 1.35)	68.9%	1.03 (0.82; 1.28)	59.2%						
4-month	1.10 (0.84; 1.44)	75.0%	1.12 (0.85; 1.46)	78.9%	1.17 (0.91; 1.51)	88.7%						
C5												
2-month	0.85 (0.67; 1.07)	91.6%	0.96 (0.76; 1.21)	64.4%	0.92 (0.73; 1.16)	75.5%	0.97 (0.77; 1.22)	60.8%				
4-month	1.10 (0.85; 1.44)	76.5%	1.12 (0.86; 1.45)	79.9%	1.17 (0.91; 1.50)	88.2%	1.14 (0.88; 1.48)	84.2%				
C6												
2-month	1.12 (0.88; 1.41)	82.0%	1.27 (1.01; 1.59)	98.0%	1.22 (0.97; 1.54)	95.4%	1.28 (1.02; 1.61)	98.5%	1.16 (0.92; 1.46)	89.0%		
4-month	1.19 (0.92; 1.54)	90.2%	1.21 (0.93; 1.56)	92.5%	1.26 (0.97; 1.63)	95.8%	1.22 (0.94; 1.59)	93.2%	1.23 (0.95; 1.61)	93.7%		

Est. – Median of the posterior distribution of incidence rate ratios with 95% compatibility intervals.
Prob. – Proportion of the posterior distribution above or below the null in the direction of the median.

Table 3 - Estimates of effects of components, including two-way interactions among components, on weekly moderate and vigorous physical activity at 2- and 4-month follow-up.

	C1		C2		C3		C4		C5		C6	
	**Est.**	**Prob.**	**Est.**	**Prob.**	**Est.**	**Prob.**	**Est.**	**Prob.**	**Est.**	**Prob.**	**Est.**	**Prob.**
**2-month**	9.34 (-25.6; 45.5)	69.7%	-5.73 (-41.9; 29.5)	63.0%	35.8 (-0.41; 71.9)	97.4%	17.1 (-19.2; 54.2)	82.2%	41.2 (5.04; 77.6)	98.7%	5.68 (-30.5; 41.1)	62.2%
**4-month**	28.1 (-11.3; 67.6)	92.0%	17.6 (-20.6; 56.5)	81.4%	6.63 (-33.1; 45.2)	63.0%	32.8 (-6.1; 71.8)	95.2%	3.02 (-36.3; 42.0)	56.0%	-19.2 (-57.6; 20.1)	83.6%
**Two-way interactions**												
**C2**												
**2-month**	3.29 (-46.2; 53.4)	55.2%										
**4-month**	43.5 (-10.0; 98.5)	94.3%										
**C3**												
**2-month**	47.3 (-4.6; 99.1)	96.3%	29.6 (-19.7; 79.6)	87.8%								
**4-month**	37.0 (-18.1; 91.4)	90.7%	21.8 (-31.5; 76.1)	78.9%								
**C4**												
**2-month**	28.7 (-23.9; 80.7)	86.3%	10.7 (-40.8; 62.0)	65.2%	49.5 (-0.48; 100.3)	97.4%						
**4-month**	60.1 (3.6; 116.5)	98.2%	49.7 (-6.7; 106.2)	95.9%	40.0 (-14.1; 93.8)	92.8%						
**C5**												
**2-month**	50.6 (-0.55; 101.9)	97.4%	34.8 (-16.7; 86.1)	90.9%	78.0 (28.3; 128.2)	99.9%	60.4 (11.3; 110.4)	99.1%				
**4-month**	31.5 (-23.4; 87.0)	86.6%	20.0 (-33.5; 75.5)	77.2%	8.2 (-46.4; 62.8)	61.9%	34.5 (-20.0; 89.4)	89.6%				
**C6**												
**2-month**	15.8 (-36.2; 67.3)	72.7%	-0.44 (-51.1; 50.5)	50.7%	41.8 (-10.1; 94.0)	94.2%	22.7 (-28.6; 72.9)	81.0%	46.1 (-4.5; 97.7)	96.4%		
**4-month**	10.7 (-45.5; 66.1)	64.4%	-2.12 (-57.7; 53.0)	52.8%	-12.2 (-69.8; 44.3)	66.4%	12.5 (-42.1; 66.9)	66.9%	-17.1 (-72.0; 37.3)	73.4%		

Est. – Median of the posterior distribution of mean differences with 95% compatibility intervals.
Prob. – Proportion of the posterior distribution above or below the null in the direction of the median.

Table 4 - Estimates of effects of components, including two-way interactions among components, on smoking cessation at 2- and 4-month follow-up.

	C1		C2		C3		C4		C5		C6	
	**Est.**	**Prob.**	**Est.**	**Prob.**	**Est.**	**Prob.**	**Est.**	**Prob.**	**Est.**	**Prob.**	**Est.**	**Prob.**
**2-month**	1.12 (0.35; 3.59)	57.4%	0.56 (0.17; 1.76)	84.3%	1.43 (0.43; 4.63)	72.3%	0.68 (0.21; 2.23)	74.5%	0.46 (0.14; 1.49)	90.4%	2.15 (0.67; 6.74)	90.4%
**4-month**	1.45 (0.35; 6.10)	69.2%	0.48 (0.11; 2.00)	84.3%	2.30 (0.52; 9.56)	87.3%	0.77 (0.17; 3.39)	64.0%	0.55 (0.13; 2.28)	79.6%	0.94 (0.22; 4.02)	53.0%
**Two-way interactions**												
**C2**												
**2-month**	0.51 (0.08; 2.89)	77.2%										
**4-month**	0.53 (0.06; 4.47)	72.0%										
**C3**												
**2-month**	1.83 (0.31; 11.2)	74.8%	0.81 (0.13; 4.91)	59.4%								
**4-month**	4.28 (0.49; 39.9)	90.7%	1.12 (0.12; 10.25)	53.9%								
**C4**												
**2-month**	0.61 (0.11; 3.51)	71.3%	0.46 (0.08; 2.51)	81.8%	0.86 (0.15; 4.70)	56.8%						
**4-month**	0.86 (0.11; 6.73)	55.8%	0.43 (0.06; 3.10)	80.3%	2.03 (0.26; 16.3)	74.5%						
**C5**												
**2-month**	0.54 (0.09; 2.99)	75.9%	0.23 (0.04; 1.27)	95.4%	0.69 (0.12; 3.83)	67.0%	0.27 (0.04; 1.64)	92.3%				
**4-month**	1.10 (0.14; 8.87)	54.0%	0.22 (0.02; 1.96)	91.2%	1.61 (0.21; 12.43)	67.8%	0.50 (0.06; 4.34)	73.7%				
**C6**												
**2-month**	2.47 (0.39; 15.1)	83.5%	1.30 (0.23; 7.22)	62.1%	3.18 (0.50; 21.0)	89.1%	2.00 (0.38; 10.5)	79.7%	1.05 (0.18; 5.9)	52.2%		
**4-month**	1.30 (0.15; 10.93)	59.4%	0.41 (0.05; 3.20)	80.4%	2.29 (0.26; 20.2)	77.5%	0.83 (0.11; 6.05)	57.3%	0.51 (0.06; 4.11)	73.6%		

Est. – Median of the posterior distribution of odds ratios with 95% compatibility intervals.

Prob. – Proportion of the posterior distribution above or below the null in the direction of the median.

Table 7 - Estimates of effects of components, including two-way interactions among components, on number of cigarettes smoked per week at 2- and 4-month follow-up.

	C1		C2		C3		C4		C5		C6	
	**Est.**	**Prob.**	**Est.**	**Prob.**	**Est.**	**Prob.**	**Est.**	**Prob.**	**Est.**	**Prob.**	**Est.**	**Prob.**
**2-month**	0.76 (0.48; 1.23)	86.8%	0.76 (0.47; 1.20)	88.1%	1.46 (0.90; 2.38)	94.2%	0.88 (0.54; 1.42)	70.5%	0.91 (0.58; 1.43)	65.6%	1.29 (0.81; 2.10)	85.4%
**4-month**	1.06 (0.61; 1.88)	57.6%	0.87 (0.50; 1.51)	69.8%	1.13 (0.66; 1.98)	66.2%	0.86 (0.50; 1.50)	69.5%	0.91 (0.52; 1.58)	63.3%	0.89 (0.52; 1.53)	66.0%
**Two-way interactions**												
**C2**												
**2-month**	0.57 (0.30; 1.09)	95.5%										
**4-month**	0.93 (0.44; 1.96)	57.0%										
**C3**												
**2-month**	1.25 (0.63; 2.55)	74.1%	1.14 (0.58; 2.21)	64.0%								
**4-month**	1.40 (0.64; 3.23)	79.8%	1.03 (0.48; 2.23)	53.1%								
**C4**												
**2-month**	0.70 (0.38; 1.30)	87.3%	0.66 (0.33; 1.32)	88.1%	1.35 (0.74; 2.50)	84.0%						
**4-month**	0.90 (0.43; 1.87)	60.9%	0.75 (0.33; 1.64)	76.0%	1.01 (0.49; 2.12)	50.7%						
**C5**												
**2-month**	0.69 (0.35; 1.37)	86.3%	0.69 (0.35; 1.35)	86.6%	1.35 (0.70; 2.60)	81.5%	0.81 (0.42; 1.57)	73.4%				
**4-month**	0.90 (0.39; 2.12)	59.5%	0.78 (0.35; 1.74)	72.4%	1.09 (0.51; 2.33)	58.5%	0.76 (0.34; 1.69)	75.4%				
**C6**												
**2-month**	0.98 (0.48; 1.97)	53.0%	0.98 (0.52; 1.87)	53.0%	1.90 (0.91; 4.10)	95.6%	1.11 (0.56; 2.21)	61.5%	1.16 (0.61; 2.23)	67.2%		
**4-month**	0.91 (0.41; 2.00)	59.4%	0.80 (0.38; 1.65)	72.8%	1.09 (0.48; 2.48)	57.7%	0.76 (0.35; 1.67)	75.6%	0.76 (0.35; 1.64)	76.1%		

Est. – Median of the posterior distribution of incidence rate ratios with 95% compatibility intervals.
Prob. – Proportion of the posterior distribution above or below the null in the direction of the median.

Table 8 - Estimates of effects of components, including two-way interactions among components, on candy and snacks at 2- and 4-month follow-up.

	C1		C2		C3		C4		C5		C6	
	**Est.**	**Prob.**	**Est.**	**Prob.**	**Est.**	**Prob.**	**Est.**	**Prob.**	**Est.**	**Prob.**	**Est.**	**Prob.**
**2-month**	1.00 (0.90; 1.12)	52.7%	1.01 (0.91; 1.14)	59.0%	0.94 (0.83; 1.05)	88.1%	0.95 (0.85; 1.07)	79.1%	1.00 (0.89; 1.12)	50.5%	1.09 (0.98; 1.22)	93.6%
**4-month**	0.92 (0.81; 1.05)	88.9%	1.00 (0.88; 1.14)	51.6%	0.92 (0.81; 1.04)	91.2%	0.96 (0.85; 1.09)	72.4%	1.12 (0.99; 1.27)	96.1%	1.07 (0.95; 1.22)	85.8%
**Two-way interactions**												
**C2**												
**2-month**	1.02 (0.87; 1.20)	59.6%										
**4-month**	0.92 (0.77; 1.10)	81.2%										
**C3**												
**2-month**	0.94 (0.80; 1.11)	78.0%	0.95 (0.81; 1.11)	74.4%								
**4-month**	0.85 (0.71; 1.01)	96.8%	0.92 (0.77; 1.10)	82.0%								
**C4**												
**2-month**	0.96 (0.82; 1.12)	70.3%	0.97 (0.83; 1.14)	65.3%	0.89 (0.76; 1.05)	92.0%						
**4-month**	0.89 (0.74; 1.06)	90.2%	0.96 (0.81; 1.15)	65.8%	0.88 (0.74; 1.05)	91.8%						
**C5**												
**2-month**	1.01 (0.86; 1.18)	53.0%	1.02 (0.87; 1.19)	58.3%	0.94 (0.80; 1.10)	78.4%	0.96 (0.82; 1.12)	70.0%				
**4-month**	1.03 (0.87; 1.23)	64.4%	1.12 (0.94; 1.34)	89.9%	1.03 (0.86; 1.22)	61.0%	1.08 (0.90; 1.29)	78.8%				
**C6**												
**2-month**	1.10 (0.93; 1.29)	86.6%	1.11 (0.95; 1.30)	89.5%	1.02 (0.87; 1.20)	58.7%	1.05 (0.89; 1.23)	71.2%	1.09 (0.93; 1.28)	86.2%		
**4-month**	0.99 (0.83; 1.18)	54.4%	1.08 (0.90; 1.28)	79.3%	0.98 (0.82; 1.17)	58.5%	1.03 (0.86; 1.23)	62.3%	1.21 (1.01; 1.44)	97.7%		

Est. – Median of the posterior distribution of incidence rate ratios with 95% compatibility intervals.
Prob. – Proportion of the posterior distribution above or below the null in the direction of the median.

Table 9 - Estimates of effects of components, including two-way interactions among components, on perceived stress at 2- and 4-month follow-up.

	C1		C2		C3		C4		C5		C6	
	**Est.**	**Prob.**	**Est.**	**Prob.**	**Est.**	**Prob.**	**Est.**	**Prob.**	**Est.**	**Prob.**	**Est.**	**Prob.**
**2-month**	-0.18<br>(-0.51; 0.15)	85.4%	0.07<br>(-0.25; 0.39)	65.8%	-0.01<br>(-0.33; 0.31)	52.3%	-0.14<br>(-0.46; 0.18)	80.3%	0.07<br>(-0.25; 0.39)	67.0%	-0.08<br>(-0.41; 0.24)	68.2%
**4-month**	-0.14<br>(-0.49; 0.22)	77.5%	-0.03<br>(-0.39; 0.32)	56.6%	-0.06<br>(-0.42; 0.29)	64.2%	-0.35<br>(-0.71; 0.01)	97.1%	-0.05<br>(-0.40; 0.31)	60.0%	0.004<br>(-0.35; 0.36)	50.8%
**Two-way interactions**												
**C2**												
**2-month**	-0.10<br>(-0.56; 0.35)	67.3%										
**4-month**	-0.18<br>(-0.68; 0.32)	75.8%										
**C3**												
**2-month**	-0.18<br>(-0.64; 0.28)	78.0%	0.06<br>(-0.40; 0.51)	59.5%								
**4-month**	-0.19<br>(-0.68; 0.31)	77.2%	-0.08<br>(-0.57; 0.41)	62.3%								
**C4**												
**2-month**	-0.31<br>(-0.76; 0.16)	90.2%	-0.07<br>(-0.54; 0.39)	62.2%	-0.17<br>(-0.62; 0.29)	76.4%						
**4-month**	-0.48<br>(-0.99; 0.04)	96.6%	-0.36<br>(-0.88; 0.15)	92.2%	-0.41<br>(-0.92; 0.09)	94.8%						
**C5**												
**2-month**	-0.10<br>(-0.55; 0.36)	66.4%	0.13<br>(-0.33; 0.58)	71.1%	0.06<br>(-0.40; 0.54)	60.6%	-0.07<br>(-0.52; 0.39)	61.2%				
**4-month**	-0.19<br>(-0.68; 0.30)	77.0%	-0.08<br>(-0.57; 0.42)	61.3%	-0.11<br>(-0.61; 0.38)	66.6%	-0.38<br>(-0.88; 0.12)	93.1%				
**C6**												
**2-month**	-0.25<br>(-0.71; 0.22)	85.8%	-0.012<br>(-0.46; 0.44)	52.0%	-0.08<br>(-0.54; 0.37)	63.9%	-0.21<br>(-0.67; 0.25)	81.8%	-0.003<br>(-0.45; 0.45)	50.6%		
**4-month**	-0.13<br>(-0.63; 0.38)	69.8%	-0.024<br>(-0.52; 0.48)	53.7%	-0.07<br>(-0.56; 0.45)	60.3%	-0.34<br>(-0.84; 0.17)	90.7%	-0.04<br>(-0.54; 0.46)	56.0%		

Est. – Median of the posterior distribution of mean differences with 95% compatibility intervals.
Prob. – Proportion of the posterior distribution above or below the null in the direction of the median.

Table 10 - Estimates of effects of components, including two-way interactions among components, on body mass index at 2- and 4-month follow-up.

	C1		C2		C3		C4		C5		C6	
	**Est.**	**Prob.**	**Est.**	**Prob.**	**Est.**	**Prob.**	**Est.**	**Prob.**	**Est.**	**Prob.**	**Est.**	**Prob.**
**2-month**	0.02 (-0.15; 0.19)	58.4%	-0.02 (-0.19; 0.16)	59.1%	-0.07 (-0.24; 0.10)	77.7%	-0.01 (-0.19; 0.16)	56.0%	0.18 (0.002; 0.35)	97.6%	0.16 (-0.01; 0.34)	96.8%
**4-month**	0.13 (-0.06; 0.32)	91.2%	-0.003 (-0.19; 0.19)	51.2%	0.06 (-0.13; 0.25)	73.6%	0.10 (-0.09; 0.29)	84.3%	0.11 (-0.09; 0.30)	86.8%	0.001 (-0.19; 0.19)	50.4%
**Two-way interactions**												
**C2**												
**2-month**	0.01 (-0.24; 0.25)	51.5%										
**4-month**	0.12 (-0.15; 0.40)	81.6%										
**C3**												
**2-month**	-0.05 (-0.30; 0.20)	65.7%	-0.08 (-0.32; 0.16)	75.0%								
**4-month**	0.191 (-0.075; 0.46)	91.8%	0.073 (-0.193; 0.341)	70.5%								
**C4**												
**2-month**	0.01 (-0.24; 0.25)	52.3%	-0.03 (-0.28; 0.22)	59.9%	-0.09 (-0.34; 0.16)	75.9%						
**4-month**	0.22 (-0.05; 0.49)	94.3%	0.10 (-0.18; 0.37)	75.5%	0.16 (-0.12; 0.43)	86.9%						
**C5**												
**2-month**	0.19 (-0.06; 0.44)	93.5%	0.16 (-0.09; 0.40)	89.1%	0.11 (-0.14; 0.36)	80.6%	0.17 (-0.08; 0.41)	90.9%				
**4-month**	0.24 (-0.03; 0.51)	96.0%	0.11 (-0.16; 0.38)	78.4%	0.17 (-0.10; 0.44)	89.9%	0.20 (-0.07; 0.47)	92.8%				
**C6**												
**2-month**	0.18 (-0.07; 0.43)	92.2%	0.14 (-0.11; 0.40)	87.2%	0.10 (-0.15; 0.35)	77.4%	0.15 (-0.10; 0.39)	88.6%	0.34 (0.09; 0.59)	99.6%		
**4-month**	0.14 (-0.14; 0.41)	83.5%	-0.01 (-0.28; 0.26)	52.4%	0.06 (-0.20; 0.34)	67.8%	0.09 (-0.18; 0.37)	75.2%	0.11 (-0.16; 0.38)	78.4%		

Est. – Median of the posterior distribution of mean differences with 95% compatibility intervals.
Prob. – Proportion of the posterior distribution above or below the null in the direction of the median
