# Supplementary material for: Effectiveness of the Components of a Digital Multiple Health Behavior Intervention Among University Students (Buddy): Factorial Randomized Trial
Source: J Med Internet Res. 2026 Mar 9;28:e88884. doi: 10.2196/88884 (PMC13010081; doi:10.2196/88884)
Supplement: Multimedia Appendix 9 [file jmir_v28i1e88884_app9.pdf]

APPENDIX H – ESTIMATES OF EFFECTS OF NUMBER OF COMPONENTS

In this appendix, post-hoc exploratory analyses are presented which estimate the effects of number of components participants had access to on primary and secondary outcomes. Analyses with complete case and imputed data are presented in Table 1 through Table 10.

In summary, the relatively strongest evidence for effects was found for increasing the module count with regards to fruit and vegetables and moderate to weekly physical activity at the 2-month follow-up, with somewhat weaker evidence for the same at 4-months. There was also relatively strong evidence for a small increase in BMI at 4-months with increased module count.

TOTAL WEEKLY ALCOHOL CONSUMPTION

Table 1 - Estimates of effects of number of components on total weekly alcohol consumption.

	Est.	Prob.
**Complete-case**		
2-month	0.94 (0.83; 1.07)	81.5%
4-month	0.95 (0.82; 1.10)	75.7%
**Imputed data**		
2-month	0.95 (0.83; 1.10)	74.7%
4-month	0.92 (0.80; 1.07)	86.2%

Est. – Median of the posterior distribution of incidence rate ratios with 95% compatibility intervals.
Prob. – Proportion of the posterior distribution above or below the null in the direction of the median.

HEAVY EPISODIC DRINKING

Table 2 - Estimates of effects of number of components on heavy episodic drinking.

	Est.	Prob.
**Complete-case**		
2-month	0.96 (0.90; 1.02)	91.2%
4-month	1.05 (0.98; 1.13)	91.1%
**Imputed data**		
2-month	0.97 (0.91; 1.04)	83.1%
4-month	1.04 (0.96; 1.12)	81.9%

Est. – Median of the posterior distribution of incidence rate ratios with 95% compatibility intervals.
Prob. – Proportion of the posterior distribution above or below the null in the direction of the median.

DAILY PORTIONS OF FRUIT AND VEGETABLES

Table 3 - Estimates of effects of number of components on daily portions of fruit and vegetables.

	Est.	Prob.
**Complete-case**		
2-month	0.05 (0.001; 0.11)	97.6%
4-month	0.04 (-0.02; 0.1)	90.7%
**Imputed data**		
2-month	0.06 (0.003; 0.11)	98.1%
4-month	0.03 (-0.04; 0.1)	80.7%

Est. – Median of the posterior distribution of mean differences with 95% compatibility intervals.
Prob. – Proportion of the posterior distribution above or below the null in the direction of the median.

SUGARY DRINKS

Table 4 - Estimates of effects of number of components on weekly sugary drinks consumption.

	Est.	Prob.
**Complete-case**		
2-month	1.02 (0.95; 1.09)	69.2%
4-month	1.08 (1.00; 1.16)	97.6%
**Imputed data**		
2-month	1.02 (0.95; 1.09)	66.3%
4-month	1.04 (0.97; 1.13)	86.4%

Est. – Median of the posterior distribution of incidence rate ratios with 95% compatibility intervals.
Prob. – Proportion of the posterior distribution above or below the null in the direction of the median.

MODERATE AND VIGOROUS PHYSICAL ACTIVITY

Table 5 - Estimates of effects of number of components on weekly moderate and vigorous physical activity.

	Est.	Prob.
**Complete-case**		
2-month	17.5 (2.9; 32.2)	99.0%
4-month	11.8 (-3.8; 27.1)	93.1%
**Imputed data**		
2-month	15.9 (0.46; 31.6)	97.8%
4-month	4.4 (-11.5; 20.8)	70.3%

Est. – Median of the posterior distribution of mean differences with 95% compatibility intervals.
Prob. – Proportion of the posterior distribution above or below the null in the direction of the median.

SMOKING CESSATION

Table 6 - Estimates of effects of number of components on smoking cessation.

	Est.	Prob.
**Complete-case**		
2-month	0.87 (0.52; 1.42)	71.1%
4-month	0.89 (0.50; 1.53)	66.1%
**Imputed data**		
2-month	0.99 (0.60; 1.60)	51.7%
4-month	0.97 (0.56; 1.65)	54.4%
**Est.** – Median of the posterior distribution of odds ratios with 95% compatibility intervals. **Prob.** – Proportion of the posterior distribution above or below the null in the direction of the median.		

CIGARETTES SMOKED PER WEEK

Table 7 - Estimates of effects of number of components on number of cigarettes smoker per week.

	Est.	Prob.
**Complete-case**		
2-month	0.96 (0.80; 1.16)	66.7%
4-month	0.95 (0.76; 1.17)	69.7%
**Imputed data**		
2-month	0.98 (0.81; 1.19)	59.6%
4-month	1.03 (0.83; 1.28)	61.3%
**Est.** – Median of the posterior distribution of incidence rate ratios with 95% compatibility intervals. **Prob.** – Proportion of the posterior distribution above or below the null in the direction of the median.		

CANDY AND SNACKS

Table 8 - Estimates of effects of number of components on candy and snacks.

	Est.	Prob.
**Complete-case**		
2-month	1.00 (0.95; 1.05)	54.3%
4-month	1.00 (0.95; 1.05)	57.5%
**Imputed data**		
2-month	1.00 (0.95; 1.05)	54.2%
4-month	0.99 (0.94; 1.04)	63.1%
**Est.** – Median of the posterior distribution of incidence rate ratios with 95% compatibility intervals. **Prob.** – Proportion of the posterior distribution above or below the null in the direction of the median.		

STRESS

Table 9 - Estimates of effects of number of components on stress.

	Est.	Prob.
**Complete-case**		
2-month	-0.04 (-0.18; 0.09)	74.2%
4-month	-0.10 (-0.25; 0.05)	90.9%
**Imputed data**		
2-month	-0.05 (-0.18; 0.09)	75.0%
4-month	-0.11 (-0.25; 0.04)	91.5%

Est. – Median of the posterior distribution of mean differences with 95% compatibility intervals.
Prob. – Proportion of the posterior distribution above or below the null in the direction of the median.

BODY MASS INDEX

Table 10 - Estimates of effects of number of components on body mass index.

	Est.	Prob.
**Complete-case**		
2-month	0.04 (-0.03; 0.12)	88.3%
4-month	0.07 (-0.01; 0.14)	94.9%
**Imputed data**		
2-month	0.04 (-0.03; 0.11)	86.2%
4-month	0.06 (-0.02; 0.13)	94.0%

Est. – Median of the posterior distribution of mean differences with 95% compatibility intervals.
Prob. – Proportion of the posterior distribution above or below the null in the direction of the median.
